# *Passer*, a highly active transposon from a fish genome, as a potential new robust genetic manipulation tool

**DOI:** 10.1093/nar/gkad005

**Published:** 2023-01-23

**Authors:** Saisai Wang, Bo Gao, Csaba Miskey, Zhongxia Guan, Yatong Sang, Cai Chen, Xiaoyan Wang, Zoltán Ivics, Chengyi Song

**Affiliations:** College of Animal Science & Technology, Yangzhou University, Yangzhou, Jiangsu, 225009, China; College of Animal Science & Technology, Yangzhou University, Yangzhou, Jiangsu, 225009, China; Division of Medical Biotechnology, Paul Ehrlich Institute, D-63225 Langen, Germany; College of Animal Science & Technology, Yangzhou University, Yangzhou, Jiangsu, 225009, China; College of Animal Science & Technology, Yangzhou University, Yangzhou, Jiangsu, 225009, China; College of Animal Science & Technology, Yangzhou University, Yangzhou, Jiangsu, 225009, China; College of Animal Science & Technology, Yangzhou University, Yangzhou, Jiangsu, 225009, China; Division of Medical Biotechnology, Paul Ehrlich Institute, D-63225 Langen, Germany; College of Animal Science & Technology, Yangzhou University, Yangzhou, Jiangsu, 225009, China

## Abstract

The discovery of new, active DNA transposons can expand the range of genetic tools and provide more options for genomic manipulation. In this study, a bioinformatics analysis suggested that *Passer* (*PS*) transposons, which are members of the *pogo* superfamily, show signs of recent and current activity in animals and may be active in some species. Cell-based transposition assays revealed that the native *PS* transposases from *Gasterosteus aculeatus* and *Danio rerio* displayed very high activity in human cells relative to the *Sleeping Beauty* transposon. A typical overproduction inhibition phenomenon was observed for *PS*, and transposition capacity was decreased by ∼12% with each kilobase increase in the insertion size. Furthermore, *PS* exhibited a pronounced integration preference for genes and their transcriptional regulatory regions. We further show that two domesticated human proteins derived from *PS* transposases have lost their transposition activity. Overall, *PS* may represent an alternative with a potentially efficient genetic manipulation tool for transgenesis and mutagenesis applications.

## INTRODUCTION

Three major types of DNA transposons, namely the classic cut-and-paste, peel-and-paste and self-replication transposons, have been recognized based on the transposition mechanism (1,2). According to the conserved catalytic domain of transposases, harbouring an acidic amino acid triad (DDD or DDE) and playing an important role in catalysing the transposition reaction (3), the cut-and-paste transposons can be subdivided into ∼20 superfamilies, e.g. *Tc1/mariner*, *Zator*, *Merlin*, *PIF/Harbinger*, *hAT* (*hobo/Ac/Tam3*), *Kolobok*, *Novosib*, *piggyBac*,*MULE* (*Mutator-like element*), *P element*, *Sola1*, *Sola2*, *Sola3*, *CMC* (*CACTA*, *Mirage* and *Chapaev*), *Transib*, *Academ* and *Ginger* (3,4). The *pogo* (DD × D) elements containing variable residues between the second and third Asp (D), which are a group of *IS630-Tc1-mariner* (*ITm*) transposons that were previously classified into the *Tc1/mariner* superfamily, have been suggested as a separate superfamily based on a recent large-scale phylogenetic analysis (4,5). High diversity has been observed within the *pogo* superfamily, and six distinct families, i.e. *Mover*, *Passer*, *Tigger*, *pogoR*, *Lemi* and *Fot/Fot-like*, were established that display a differential taxonomic distribution and structure organization (4). The *Passer* (*PS*) family, which is distributed extensively in both vertebrates and invertebrates, is characterized by the highly conserved catalytic motif (DD35D) of transposases, containing 35 residues between the second and third ‘D’ of the triad signatures, and the presence of short terminal inverted repeats (TIRs) (4).

The cut-and-paste transposition reaction requires the recognition of two TIRs by the transposase, to cleave and release the DNA transposon from the original site, followed by the integration of the excised transposon into a new location, executed by the same transposase (6). Because of their inherent capacity for insertion into DNA, cut-and-paste transposons can be developed into powerful tools for genome manipulations, such as gene discovery applications in functional genomics and gene delivery systems for transgenesis and gene therapy (7–9). Cut-and-paste transposons, in particular, have demonstrated great potential as delivery vectors in germline transgenesis and insertional mutagenesis (7,8), with *ZB* displaying an efficient enhancer trapping in zebrafish and mice (10). Furthermore, the application of cut-and-paste transposons in gene therapy has been extensively reported and shows great potential in cancer immune therapy (11–13).

Various cut-and-paste transposons have been identified or developed into efficient genetic tools. Some naturally active insertion sequences or transposons identified in bacteria, such as *ISY100*, Tn*10* and Tn*5* (14–16), have been reported as being efficient gene delivery tools in prokaryotic organisms, whereas others demonstrated wide applications in eukaryotes, such as *P* elements (17), *Sleeping Beauty* (*SB*) (18), *piggyBac* (*PB*) (19), *Minos* (20), *Mos1* (21), *TcBuster* (22) and *Tol2* (23). In particular, *ZB* (from zebrafish) and *SB* from the *Tc1*/*mariner* superfamily, *PB* from the *piggyBac* superfamily and *Tol2* from the *hAT* superfamily have been successfully used in vertebrate transgenesis and mutagenesis (10,18,24–25). The biological characteristics of transposons, including their structure organization, cargo capacity and genome-wide integration preference, such as in *PB*, *SB*, *Tol2* and *ZB*, are substantially different, and these transposons therefore offer advantages in different applications (8,10,24,26–27)

Here, to expand the range of genetic tools for differential applications in life sciences, we reinvestigated the distribution and evolution dynamics of *PS* transposons in detail and evaluated the transposition activities of *PS* transposons from three fish genomes (*Gasterosteus aculeatus*, *Maylandia zebra* and *Danio rerio*) in human cells. A highly active *PS* element from *G. aculeatus* was obtained, and its cargo capacity and genome-wide integration preference in the human genome were characterized; moreover, we found that two genes in the human genome that have evolved from *PS* transposases (4), i.e. *POGK* containing a Kruppel-associated box (KRAB) domain and *POGZ* containing a zinc finger (ZNF) domain, had lost their transposition activity.

## MATERIALS AND METHODS

### *PS* transposon mining

To assess the distribution of *PS* transposons in genomes, *PS* transposases that were mined in related species were used as references to search the whole-genome shotgun contig database (WGS) at the NCBI using TblastN with a value of 1^e-100^. The *PS* transposons were manually determined to exist in a species when the catalytic domain (DD35D) was retrieved; subsequently, related transposases were used as queries to detect more specific transposons. Moreover, significant hits were extracted with 2000 bp flanking sequences, which were aligned to determine their boundaries [target site duplications (TSDs) and TIRs] using ClustalW within BioEdit (28,29). Then, all hits obtained from the RefSeq genome database (if no species was available, the WGS database was used) with >40% coverage in length and >80% identity were used to estimate copy numbers. In addition, the consensus sequence was constructed using DAMBE (30) if there were more than five intact copies in each species. In species with fewer than five full-length copies, the representative sequence was the intact one that contained the complete TIR and encoded an intact transposase protein (>300 amino acids, containing an intact DNA-binding domain, abbreviated as DBD, and DDE domains). The consensus or representative sequence was used for further analysis. The secondary structure of the transposase was predicted using the PSIPRED program (http://bioinf.cs.ucl.ac.uk/psipred/) (31). Putative nuclear localization sequence (NLS) motifs were predicted using the WOLFPSRT program (https://wolfpsort.hgc.jp/) (32). The protein domains were identified using hidden Markov models with the online hmmscan web server (https://www.ebi.ac.uk/Tools/hmmer/search/hmmscan) (33).

### Phylogenetic inference and evolutionary dynamics analysis

To define the evolutionary history of these families, the conserved DDD domains of *PS* transposases were aligned with those of representative families from the *Tc1*/*mariner* and *pogo* superfamilies using MAFFT v.7.310 (4,34). The phylogenetic tree was inferred based on the conserved DDE/D domain (∼150 amino acids) using the maximum likelihood method with IQ-TREE (35). The best-fit model was selected by ModelFinder embedded in the IQ-TREE program, and the reliability of the maximum likelihood trees was estimated using the ultrafast bootstrap approach with 1000 replicates (36). The activities (evolution dynamics) of *PS* in genomes were deduced using the Kimura two-parameter distance (K divergence) (37), which was computed using the calcDivergenceFromAlign.pl package from RepeatMasker (38).

### Plasmids used for the verification of the transpositional activity of the *PS* transposon system

The transposase open reading frames (ORFs) of *PS* were amplified by nested polymerase chain reaction (PCR) from *D. rerio*, *G. aculeatus* and *M. zebra* genomic DNA samples, respectively, using primers designed according to the *PS* gene sequences (Supplementary File S1). The PCR products were cloned into the pLB vector using a Lethal Based Fast Cloning Kit (TIANGEN, Beijing, China) and sequenced at the Tsingke Biotechnology Company. PSTIR consensus sequences from teleost fish and the *G. aculeatus* genomes were obtained by annealing oligos (PS-TIR-F/PS-TIR-R, GAAC-TIR-F/GAAC-TIR-R), then cloned into the pLB vector and sequenced. The obtained vectors were named pPSTIR and pGaacTIR. All primers and oligos used here are listed in Supplementary Table S2.

For the assay of the transposition characteristics of the transposons, two plasmid systems were used. The donor plasmids, pPS-PGK-Neo and pGaacPS-PGK-Neo, were prepared by inserting the PGK-Neo cassette into pPSTIR and pGaacTIR through the NruI site (New England Biolabs, USA), respectively. The helper plasmids expressing transposases were generated by inserting the transposase ORF via the BamHI and XhoI sites (Takara, Japan) into the modified pcDNA3.0, in which the *Neo* gene expression cassette was removed through the Psil sites (Takara, Japan). The resulting recombinant vectors were termed pcDNA3.9-DarePSase, pcDNA3.9-GaacPSase, pcDNA3.9-MazePSase1, pcDNA3.9-MazePSase2 and pcDNA3.9-MazePSase3. As described previously (39), the TIR and transposase sequences (SB100X) from the *SB* system were subcloned from pUC19SBneo and pCSBNpA, respectively, and inserted into the same donor and helper vectors of the *PS* system as a control. These plasmids were named pSB-PGK-Neo and pcDNA3.9-SB100X, respectively. Based on the transposition activity assay, the optimized *PS* system, PSTIR and GaacPSase were subcloned into the same donor and helper vectors as pUC19SBneo and pCSBNpA for overproduction inhibition assay. Briefly, the PS-PGK-Neo cassette was obtained by PCR using the primers UC19PSneo-F and UC19PSneo-R (listed in Supplementary Table S2) and inserted into pUC19SBneo via the KpnI and XbaI sites (Takara, Japan). The GaacPSase ORF was obtained by PCR using the primers listed in Supplementary Table S2 (PSase-F and PSase-R), and inserted into pCSBNpA via XhoI and NotI (Takara, Japan). After sequencing, the correct clones were named pUC19PSneo and pCPSNpA. Here, the highly active SB100X transposase was applied in all cell tests as controls. All vector sequences are listed in Supplementary File S4.

### Plasmids used for the analysis of the cargo capacity of PS transposons

For cargo capacity assay, serial donor plasmids harbouring different sized fragments were constructed. Briefly, the donor plasmid pPS-PGK-NEO harbouring a 1.6 kb fragment was cut by EcoRI and ligated with 1.5, 3.5 and 6.9 kb fragments of the λ-phage genome, respectively, between poly(A) and the right TIR, to give pPS-PGK-NEO/3.1/5.1/8.5. To fill the donor plasmid size, 5.5, 3.5 and 6.9 kb fragments of λ-phage DNA (as stuffer material) were cloned into pPS-PGK-NEO/1.6/3.1/5.1 via the HindIII site outside transposon TIRs (40). All stuffers were PCR amplified from λ genomic DNA. The primers used are listed in Supplementary Table S2. In the cargo capacity assay, three repetitions were performed in each group. All vector sequences are listed in Supplementary File S4.

### Cell culture and transfection

HeLa and HepG2 cells (ATCC, USA) were maintained in Dulbecco’s modified Eagle’s medium (DMEM; Gibco, USA) with 10% foetal bovine serum (FBS; Gibco, USA) and 1% penicillin–streptomycin (Gibco, USA) in an incubator at 37°C with 5% CO_2_. Transfection was performed according to the technical manual (FuGENE® HD Transfection Reagent, Promega, USA). Briefly, 3 × 10^5^ cells are seeded onto 6-well plates 1 day before transfection to achieve ∼80% confluence of the cells on the day of transfection. Donor plasmids and transposase-expressing helper plasmids were co-transfected using FuGENE^®^ HD transfection reagent (Promega, USA) with a reagent to DNA ratio of 3:1. In the transfection assay, three repetitions were performed in each group.

### Transposition and overproduction inhibition assay

The transpositional activities of *PS* from different fishes were assessed as described above. Briefly, 500 ng of donor plasmids and 500 ng of helper plasmids were co-transfected into HeLa cells. At 48 h post-transfection, the cells were re-plated in 10 cm plates and selected in 600 μg/ml G418 medium (Gibco, USA). After 2 weeks of selection, the resistant colonies were stained with Giemsa reagent (Promega, USA) and counted using ImageJ.

Overproduction inhibition (OPI) was assayed as described previously (26). Briefly, 10 or 500 ng of donor plasmids was co-transfected with varying amounts of helper plasmids, from 0 to 1500 ng (each transfection reaction was made up to 1.5 μg with the empty vector pcDNA3.9). At 48 h post-transfection, the cells were trypsinized and re-plated into 10 cm plates (seeding 10% of the transfected cells for 10 ng of donor plasmid, and 1% of the transfected cells for 500 ng of donor plasmid), then selected in 600 μg/ml G418 medium. After 2 weeks of selection, the G418-resistant colonies were stained with Giemsa, and the colony numbers were statistically analysed using ImageJ. Three replicates of each sample in three independent experiments were prepared.

### Cell staining and counting

After 14 days of G418 selection, the colonies were stained following the Giemsa reagent manual. In brief, the cell colonies were washed with phosphate-buffered saline (PBS) and incubated in the mixture (PBS:methanol = 1:1) for 2 min at room temperature. The cell colonies were fixed for 10 min at room temperature in methanol, then incubated in Giemsa reagent for 2 min, and followed by incubating in the 10 times diluted Giemsa reagent for 2 min. Finally, the stained colonies are scanned and counted by Image J.

### Excision assay and footprint analysis

A plasmid-based excision assay was used to detect the relative excision activity of *PS* transposases according to a previous protocol (6,10). Briefly, 500 ng of donor plasmid was co-transfected with 500 ng of helper plasmid into 3 × 10^5^ HeLa cells. At 48 h post-transfection, the cells were collected and DNA was extracted using the TIANamp Genomic DNA Kit (TIANGEN, Beijing, China). About 40 ng of template DNA was used for real-time PCR with the TB Green® Premix Ex Taq™ II (Tli RNaseH Plus) Kit (TAKARA, Dalian, China) by using qTower^3^G (Germany). The primers used for the excision assay were the outside left and right TIRs. The quantitative PCR (qPCR) conditions were as follows: 95°C, 30 s; 40 cycles (95°C, 5 s; 60°C, 30 s). The relative excision activity was calculated based on ΔΔCt.

To sequence the footprint left by the transposon, the PCR excision products were cloned using primers (LB-F and LB-R) designed according to the sequence of the cloning vector (Supplementary Figure S4) and sequenced using the sequencing primer (Footprint-seq). The primers used in this assay are listed in Supplementary Table S2.

### Insertion site analysis

The insertion site libraries for Illumina sequencing were prepared as described previously (10). Briefly, genomic DNA was isolated from HepG2-resistant colonies transfected with pPS-PGK-Neo and pcDNA3.9-GaacPSase,and selected in 1 mg/ml G418 for 2 weeks. DNA samples were sonicated to an average length of 600 bp using a Covaris M220 ultrasonicator (Covaris, USA). Fragmented DNA was subjected to end repair, dA tailing and linker ligation steps. Transposon–genome junctions were then amplified by nested PCRs using two primer pairs binding to the transposon TIR and the linker, respectively. The PCR products were separated on a 1.5% ultrapure agarose gel and bands with a size range of 200–500 bp were extracted from the gel. Some of the generated products were cloned and Sanger sequenced for library verification before high-throughput sequencing on a NextSeq (Illumina) instrument using the single-end 86 bp setting. The essentially nested primers (PSnest1, PSnest2, LinkerNest1 and Linker Nest2) were used to perform nested PCR (listed in Supplementary Table S2).

The conditions and thresholds of the raw read processing and mapping parameters have been specified previously (41). In short, the raw reads were subjected to quality trimming, and the resulting reads were mapped to the hg38 human genome assembly using bowtie2 (42). The coordinates of the genic features were downloaded from the UCSC Table Browser (https://genome.ucsc.edu/cgi-bin/hg). The *allOnco* gene collection of cancer-related genes was described previously (43). Insertion site distributions around the transcription start sits (TSSs) were plotted using the genomation package (44) of the R environment (https://www.R-project.org). The HepG2 cell line-specific gene expression data were obtained from the Human Protein Atlas (https://www.proteinatlas.org/about/download). The insertion frequencies in gene groups with increasing expression levels were normalized to the length of the genes and the number of genes within the group. The HepG2-specific chromatin states based on imputed histone modification (ChIP-Seq) datasets were downloaded from the Roadmap Epigenomics Project (https://egg2.wustl.edu/roadmap/web_portal/imputed.html#chr_imp). The insertion site frequencies of *PS* and *SB* (41) within these regions were compared with a set of 100 000 computationally generated random loci in the human genome. The coordinates of PS and SB insertions in the human genome are listed in Supplementary Files S2 and S3.

### Transpositional activity of *POGK* and *POGZ* derived from *PS* transposase

To verify the functional transposition activity of the *PS* domesticated genes *POGK* and *POGZ*, total RNA was extracted from HepG2 cells and the cDNA first strand was synthesized by FastKing gDNA Dispelling RT SuperMix (TIANGEN, Beijing, China). The coding sequences (CDSs) of *POGK* and *POGZ* were amplified from cDNA by PCR using the primers POGZ-CDS-F/POGZ-CDS-R and POGK-CDS-F/POGK-CDS-R listed in Supplementary Table S2. Subsequently, the *POGK* and *POGZ* CDSs were cloned into the pLB vector using the Lethal Based Fast Cloning Kit (TIANGEN, Beijing, China), and then sequenced. The resulting correct recombinant clones were termed pLB-POGK and pLB-POGZ. Next, the *POGK* and *POGZ* CDSs were subcloned downstream of the cytomegalovirus (CMV) promoter in pT2-CMV-SB100X vector by the SacII sites. The resulting recombinant vectors were termed pT2-CMV-POGZ and pT2-CMV-POGK.

A 500 ng aliquot of pT2-CMV-POGZ or pT2-CMV-POGK as helper plasmids was co-transfected into HeLa cells with 500 ng of the donor plasmid pPS-PGK-neo. The transposition assay was performed as described above.

### Statistical analyses

The statistical significance was derived from Fisher's exact test, *P*-values are depicted as follows: ****P ≤10^–4^; ***10^–4^ <*P* ≤0.001; **0.001 <*P* ≤0.01; *0.01 <*P* ≤0.05.

## RESULTS

### Recent and current activities of *PS* transposons in multiple animal lineages

**Figure 1.**
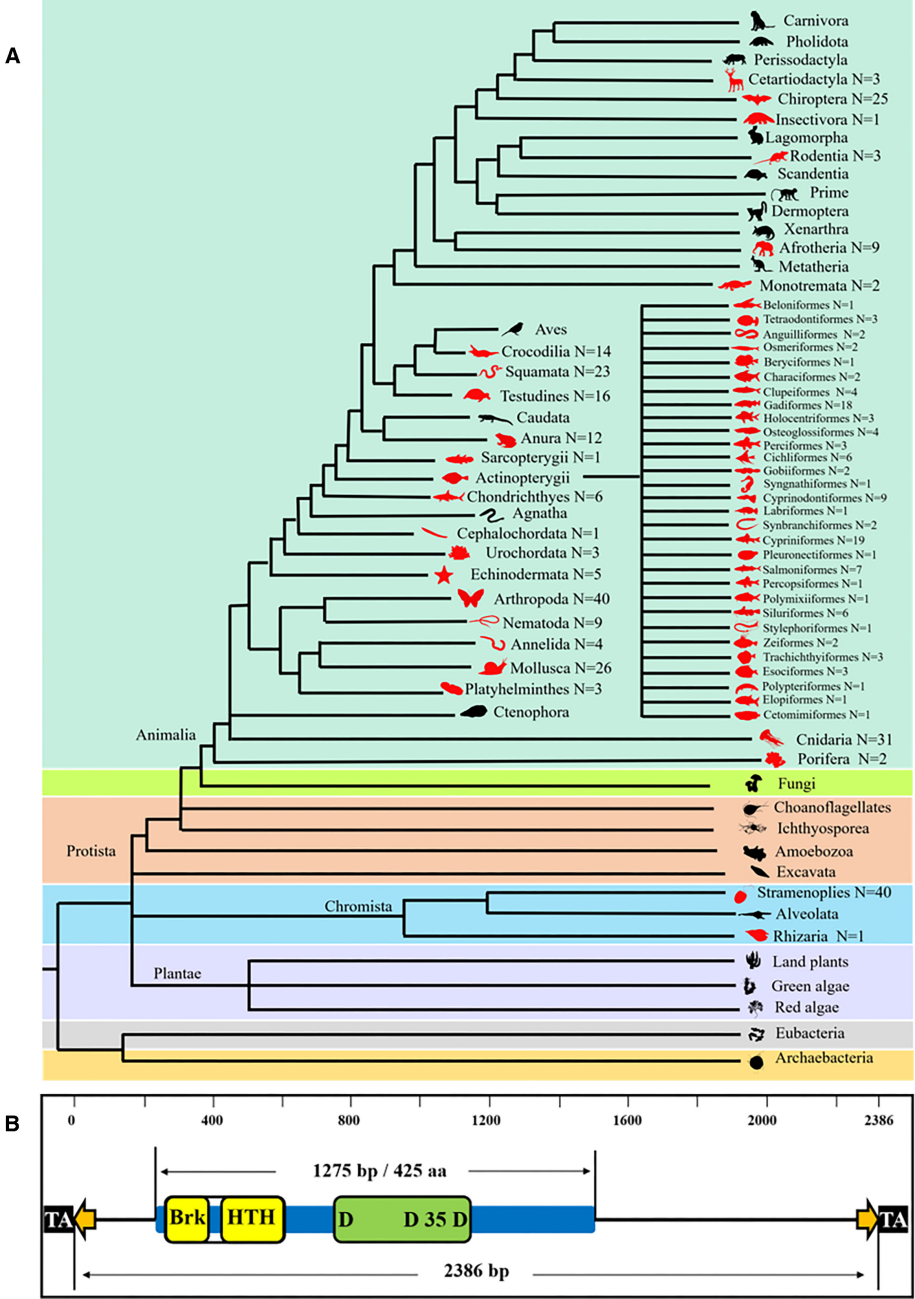
Taxonomic distribution and structure of *PS* transposons. (**A**) Taxonomic distribution of *PS* transposons in the **s**even kingdoms, indicated by different background colours. The red colour in the graph represents the presence of *PS*. N next to the animal silhouettes represents the number of *PS* transposons detected in the species of each lineage. (**B**) Structural and functional component of representative *PS* transposons of *Gasterosteus aculeatus*. The element contains a single gene encoding the transposase (blue rectangle). The black squares represent TA TSD nucleotide sites, the orange arrows represent TIRs, the yellow rectangle represents the DNA-binding domain, containing two DBD motifs (Brk and HTH), and the green rectangle represents the catalytic domain.

The taxonomic distribution of *PS* elements was reinvestigated and updated by a TBlastN search, as described in the Materials and Methods. In total, 404 *PS* transposons in 391 species, including 100 *PS* transposons in 89 species that had been identified previously (4), were obtained (Figure 1; Supplementary Table S1). All identified elements were confirmed as *PS* members by phylogenetic analysis (Supplementary Figure S1); they formed a monophyletic clade with very high bootstrap support (98%) and were distinct from other *pogo* families, including *Tigger*, *pogoR*, *Lemi*, *Mover* and *Fot* (Supplementary Figure S1A, B), as designated in the previous study (4). A wide distribution of *PS* transposons was found mainly in animals, but they also invaded the kingdom of Chromista, including one species of Rhizaria and 40 species of Stramenoplies. In the kingdom of animals, *PS* was detected in 124 species from 10 invertebrate phyla and 226 species from 13 vertebrate orders; and even in mammals, where *PS* expanded into five classes of mammals, including two species of Monotremata, nine species of Afrotheria, three species of Rodentia, one species of Insectivora, 25 species of Chiroptera and three species of Cetartiodactyla. Overall, *PS* elements invaded almost all lineages of animals, with the exception of Ctenophora, Agnatha, Caudata and Aves (Figure 1; Supplementary Table S1).

Over 200 species (50% of the total species in which *PS* was detected) harbour intact *PS* transposons in genomes. Intact *PS* transposons have a total length range of 1.6–4.0 kb, with one ORF encoding intact transposases encompassing 400–500 amino acids, flanked by TIRs and TA TSDs (Figure 1B; Supplementary Figure S2). Moreover, most *PS* transposons are characterized by very short TIRs (<30 bp), compared with highly active *ZB* (10) and *SB* (45) transposons. Most *PS* transposases are predicted to contain one DBD (harbouring two types of DBD motifs), an NLS and a catalytic domain (DD35D) (Figure 1B; Supplementary Figure S2; Supplementary Table S1).

**Figure 2.**
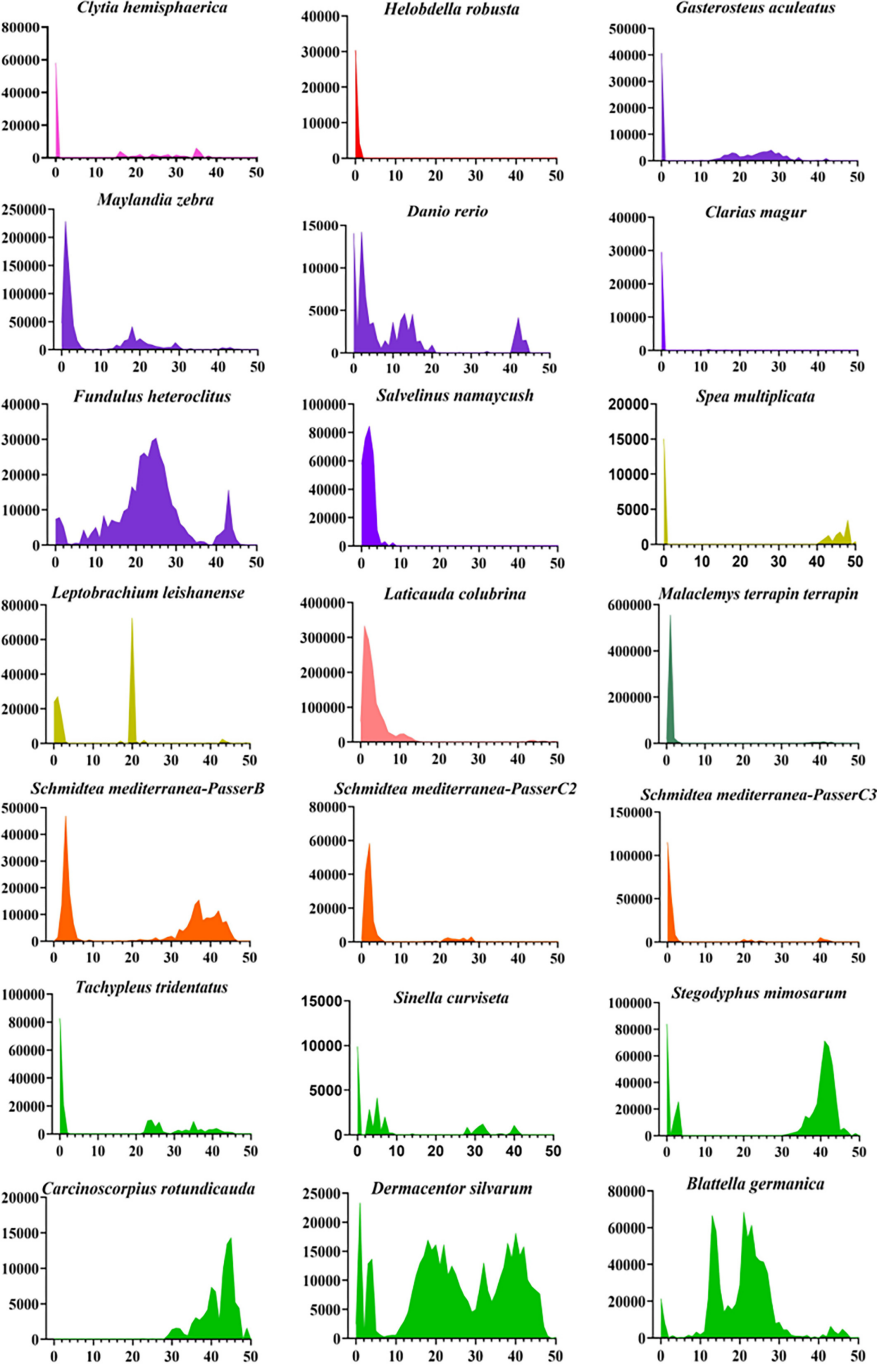
Evolutionary dynamics of *PS* transposons in animals. RepeatMasker utility scripts were used to calculate the K divergence from consensus sequences or the representative sequence. Species with <10 intact copies of *PS* in their genomes were excluded from the analysis. The *y*-axis represents the coverage (bp) of each *PS* element in the genome, and the *x*-axis indicates the Kimura divergence (%) estimate. Pink, Cnidaria; red, Annelida; purple, Actinopterygii; yellow, Anura; light pink, Squamata; atrovirens, Testudines; orange, Platyhelminthes; green, Arthropoda.

The structural features of *PS* transposons suggest that they might be currently active in multiple lineages of animals (Supplementary Table S1), with more than five intact transposon copies, indicating that the transposons contain an ORF encoding a putative transposase (including two DBD motifs and a DDE catalytic domain) flanked by TIRs, which have been detected in some genomes (Table 1). In particular, in some species, the evolutionary dynamics of *PS* (Figure 2) revealed that some *PS* copies display low levels of Kimura (K) divergence (<2%), supporting the idea that these transposons are very young insertions in genomes. The K divergence represents the insertion age of the transposon in the genome, and it is generally believed that the younger transposons display lower Kimura divergences due to lower accumulation of mutations (37). Furthermore, very high identities of transposase (>96%) and 5′ and 3′ TIR (>97%) sequences were observed for *PS* transposons in *G. aculeatus*, *D. rerio*, *Spea multiplicata*, *Clytia hemisphaerica* and *Sinella curviseta* (Table 1), indicating that *PS* in these species may be currently active. In addition, relatively low transposase identities (65–85%, Table 1) and multiple waves of amplification of *PS* were observed in some species (such as *Carcinoscorpius rotundicauda*, *Blattella germanica* and *Dermacentor silvarum in* arthropods; and *Fundulus heteroclitus*, *M. zebra* and *Salvelinus namaycush* in Actinopterygii; Figure 2), indicating that these species experienced repeat invasions of *PS*, and that current, recent and ancient copies of *PS* transposons co-exist in genomes.

**Table 1.** Summary of *PS* transposons from 21 species with more than five intact copies

Species name	Group	Total copy number	Intact copy number	Transposon length (bp)	Transposase length (amino acids)	Transposase identity (%)	5′/3′ TIR length (bp)	5′/3′ TIR identity (%)
*Clytia hemisphaerica*	Cnidaria	18	8	2888	475	96.96	27/27	100/100
*Helobdella robusta*	Annelida	16	12	1773	437	90.83	24/24	99.3/95.23
*Gasterosteus aculeatus*	Actinopterygii	16	16	2388	425	99.97	28/28	97.3/97.06
*Maylandia zebra*	Actinopterygii	127	30	1784	434	77.90	28/28	84.48/95.84
*Danio rerio*	Actinopterygii	26	7	1755	425	97.12	28/28	99.05/99.05
*Clarias magur*	Actinopterygii	17	5	2384	425	100	28/28	100/100
*Fundulus heteroclitus*	Actinopterygii	8	5	2048	427	74.94	28/28	93.88/93.52
*Salvelinus namaycush*	Actinopterygii	63	19	1819	439	83.19	18/18	96.84/95.68
*Spea multiplicata*	Anura	8	8	1877	425	99.18	27/27	96.03/100
*Leptobrachium leishanense*	Anura	14	14	1777	446	84.27	27/27	96.35/98.92
*Laticauda colubrina*	Squamata	294	83	2065	283	66.58	25/25	89.26/93.21
*Malaclemys terrapin*	Testudines	238	9	2541	450	65.76	27/27	96.97/90.8
*Schmidtea mediterranea* (*PasserB1*)	Platyhelminthes	167	7	1738	431	85.48	21/21	97.26/87.26
*Schmidtea mediterranea* (*PasserC2*)	Platyhelminthes	230	10	1697	443	84.61	28/28	97.28/78.61
*Schmidtea mediterranea* (*PasserC3*)	Platyhelminthes	271	16	1710	446	86.1	25/25	97.63/95
*Tachypleus tridentatus*	Arthropoda	130	38	2209	437	77.19	25/25	97.33/97.4
*Sinella curviseta*	Arthropoda	5	5	1975	351	99.88	25/25	100/100
*Stegodyphus mimosarum*	Arthropoda	60	34	2133	428	100	29/29	87.71/98.89
*Carcinoscorpius rotundicauda*	Arthropoda	59	14	2104	440	82.39	27/27	97.93/97.38
*Dermacentor silvarum*	Arthropoda	9	9	2249	417	83.59	28/28	100/99.24
*Blattella germanica*	Arthropoda	43	13	1743	421	90.18	32/32	99.02/97.8

Intact copy number of transposons indicates the number of full transposons containing two TIRs and intact transposases (contain two DBD motifs and a DDE catalytic domain). Transposase identity was calculated by using all intact *PS* transposase sequences in a given genome. TIR identity was calculated by comparing all TIRs (5′ and 3′) of the detected elements in a given genome; the intraelement TIR identity was calculated by comparing the 5′ TIR and 3′ TIR for each detected element.

### Highly active *PS* transposases from fish genomes

Their evolution dynamics analysis and the high number of intact copies in genomes suggest that *PS* transposons are young invasions in some fish genomes and may be highly active. To identify highly active elements, *PS* transposons from three fish genomes (*G. aculeatus*, *M. zebra* and *D. rerio*), which have a similar structure organization with a single ORF encoding transposases of 425 or 434 amino acids flanked by TIRs of 28 bp (Table 1), and their genomic DNA samples which are available in our laboratory, were selected for the evaluation of transposition activity in human cells. *PS* transposons in both *G. aculeatus* and *D. rerio* seem to be very young invasions with high transposase and TIR identities (>97%) and very low K divergences, suggesting that they might be transpositionally active, whereas *PS* transposons in *M. zebra* may represent low activity with low transposase identity (77.9%) in the genome (Figure 2; Table 1), which was used as a parallel comparison.

**Figure 3.**
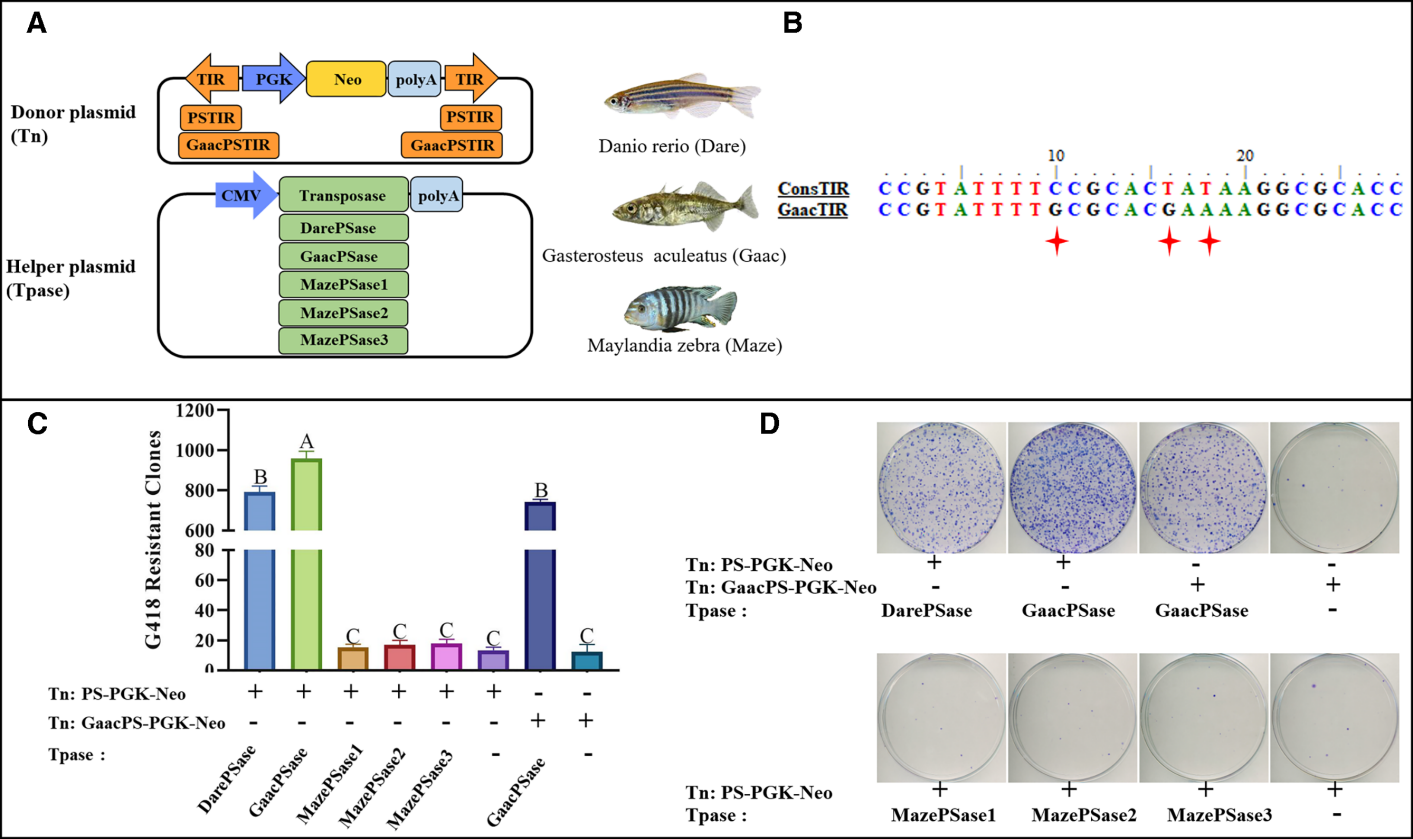
Transposition activities of *PS* elements in human cells. (**A**) Donor and helper plasmids used in human cells. Donor plasmids: the orange arrows represent transposon TIRs including PSTIR and GaacPSTIR; *PGK*, *PGK* promoter; *Neo*, neomycin resistance gene. Helper plasmids: *CMV*, *CMV* promoter; transposase, the transposase (DarePSase, GaacPSase and MazePSase) ORF. (**B**) The alignment of ConsTIR and GaacTIR. ConsTIR is constructed using PSTIR sequences from 18 teleost genomes (Supplementary Figure S3A). GaacTIR is constructed using all PSTIRs from the *G. aculeatus* genome (Supplementary Figure S3B). (**C**) Transposition activities of five *PS* transposases. Different upper case letters show a significant difference (*P* <0.01) between bars, while the same upper case letters showed no difference (*P* >0.05) between bars. (**D**) G418-resistant cell colonies stained by Giemsa.

To validate the transposition activity of *PS* from different fish genomes, a binary transposition system based on co-transfection of a donor transposon and a helper transposase plasmid was used (10,26). Two transposon donor vectors were constructed with the same *Neo* expression cassette, but different types of TIRs (Figure 3B): one type of TIR was the consensus sequence constructed using all PSTIRs from the *G. aculeatus* genome (termed GaacTIR, Supplementary Figure S3), whereas the other type of TIR (termed ConsTIR) was the consensus sequence that was constructed using PSTIR sequences from 18 teleost genomes that display a very close phylogenetic relationship in the IQ-TREE, as assessed based on the *PS* DDE domain (Supplementary Figure S1B). Three nucleotides (C/G, T/G and T/A at positions 10, 16 and 18) were different between GaacTIR and ConsTIR, and the donor plasmids harbouring ConsTIR and GaacTIR were named PS-PGK-Neo and GaacPS-PGK-Neo, respectively (Figure 3A, C). For helper plasmids, five transposase-coding sequence clones were obtained, three from the *M. zebra* (Maze) genome, one from the *D. rerio* (Dare) genome and one from the *G. aculeatus* (Gaac) genome; they were submitted for sequencing and subcloned into the expression vectors, which were termed pcDNA3.9-DarePSase, pcDNA3.9-GaacPSase, pcDNA3.9-MazePSase1, pcDNA3.9-MazePSase2 and pcDNA3.9-MazePSase3, respectively (Figure 3A).

The transposition assay showed that the transposase (GaacPSase) from the *G. aculeatus* genome displayed the highest transposition activity, followed by the *D. rerio PS* transposase (DarePSase), whereas none of the three transposase clones (MazePSase1, MazePSase2 and MazePSase3) from *M. zebra* show detectable transposition activity. In addition, we found that the donor plasmid (PS-PGK-Neo) harbouring the consensus sequence of PSTIR (ConsTIR) exhibited a significantly higher transposition activity than did the donor plasmid (GaacPS-PGK-Neo) with GaacTIR when co-transfected with the helper plasmid pcDNA3.9-GaacPSase (Figure 3C, D). Moreover, the same tendency of excision activity was observed for these transposases (Supplementary Figure S4). The group including GaacPSase and ConsTIR showed the highest transpositional activity, so we named them as the *PS* system containing a donor plasmid pPS-PGK-Neo and a helper plasmid pcDNA3.9-PSase (PSase indicates the GaacPSase and PSTIR indicates the ConsTIRs) which were used for subsequent experiments.

### *PS* displaying typical overproduction inhibition (OPI) with a transposition activity comparable with *SB*

**Figure 4.**
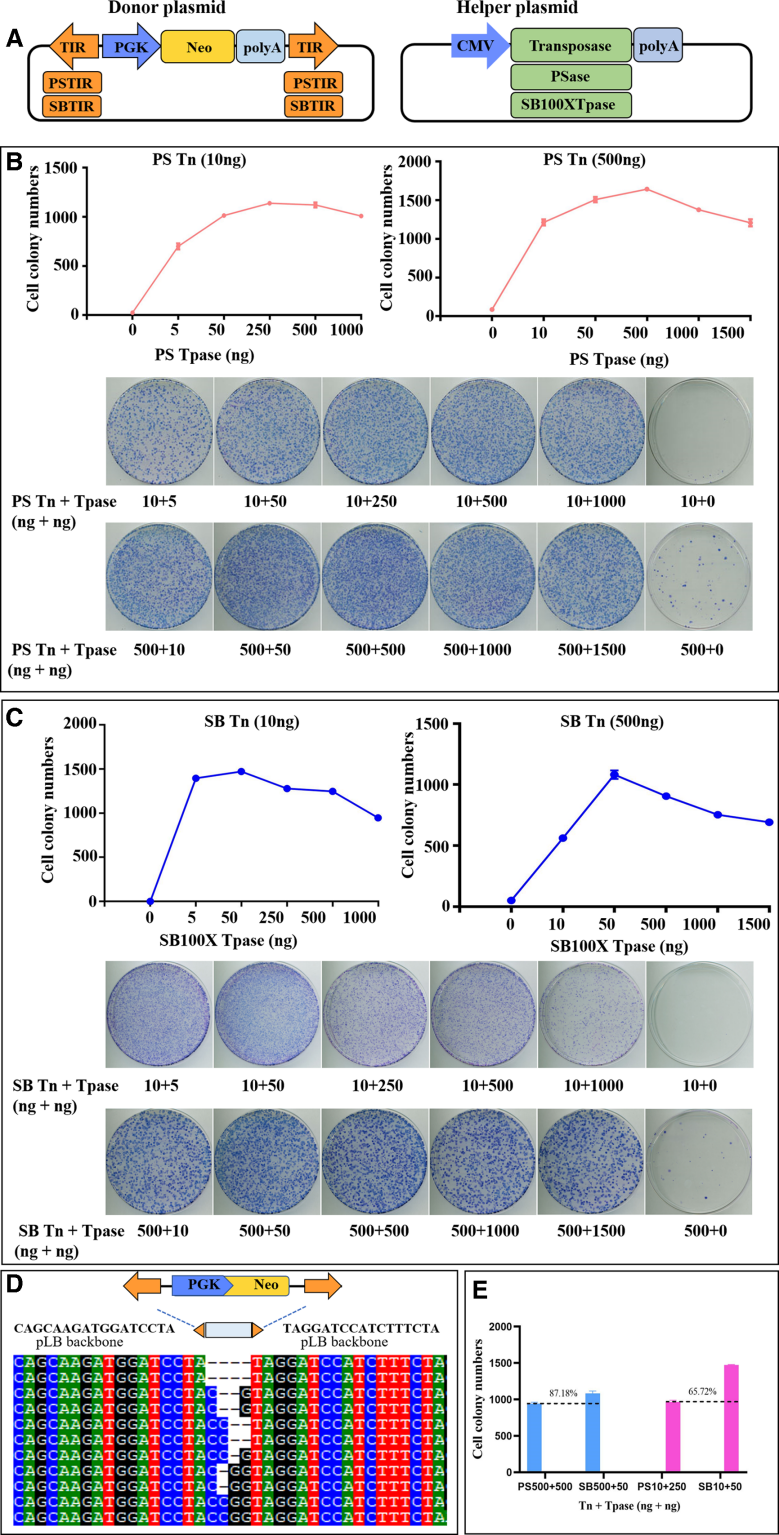
Transposition activities of *PS* and *SB* in human cells. (**A**) Donor and helper plasmids used in human cells. Donor plasmids: the arrows represent transposon TIRs, including PSTIR and SBTIR; *PGK*, *PGK* promoter; *Neo*, neomycin resistance gene. Helper plasmids: *CMV*, *CMV* promoter; transposase, the transposase (PSase, SB100X)-encoding gene. (**B**) Transposition activities of *PS* in HeLa cells co-transfected with different transposon DNA amounts (10 and 500 ng). (**C**) Transposition activities of *SB100X* in HeLa cells co-transfected with different transposon DNA amounts (10 and 500 ng). Three repeats were performed in each group. (**D**) Excision footprints of *PS*. A schematic representation of the donor is shown on top. The orange arrows indicate PSTIR and the pLB vector backbone sequences that flank the element in the donor construct. The transposon footprints are depicted at the dotted line position. (**E**) Comparative transposition activities in HeLa cells in high and low transposon DNA conditions. The errors were calculated and the plots (B and C) and the bars (E) represent the results of three independent repeats.

The putative OPI of *PS*, which is a phenomenon that is caused by the inhibition of transposition activity by excess transposase expression (26), was investigated by using the *SB* transposon system as a parallel comparison. The same backbones of donor and helper plasmids were used to minimize the difference between *PS* and *SB* systems, with the exception of transposon-specific TIRs and the transposase-coding sequences, which were different between vectors and different transposons (Figure 4A).

The OPI for *PS* and *SB* transposons was compared under low (10 ng) and high (500 ng) dosages of transposon donor plasmids. The *PS* transposon system exhibited OPI at both low and high dosages (Figure 4B). *PS* reached peak activity at 250 ng of the helper plasmid transfected at the low dose of the transposon donor plasmid (10 ng), and at 500 ng of the helper plasmid at the high dose of the donor plasmid (500 ng). Moreover, for the *SB* transposon system in this assay, *SB* also showed OPI at both low and high dosages. At the low amount of donor plasmid (10 ng), SB100X reached its peak at 50 ng of transposase plasmid transfected, while it needed 50 ng of transfected transposase plasmid to produce its highest colony number at the high dose (500 ng) of donor plasmid (Figure 4C). Neither the *PS* nor the *SB* system exhibited a plateau effect after reaching the peak in the two transposition conditions; these two systems showed a decrease in their activities, which was consistent with OPI. In contrast, a discrepancy was observed between the report by Grabundzija *et al.* (26) and our current OPI data. In the current experiment, *SB* activity is only slightly reduced by excess transposase (Figure 4C), whereas previously its activity was significantly decreased by excess transposase (26). Further analysis revealed that the discrepancy may be due to the differences in vector sequences, as a significant decrease in transposition activity with excess transposases was observed for the *SB* transposon (Supplementary Figure S5) when the same donor vector backbone constructed by Grabundzija *et al.* (26) was used. In addition, *PS* transposition generates footprints ranging from 0 to 4 bp at the excision site (Figure 4D), which is similar to that observed for *Tc1*/*mariner* transposons, such as *ZB* (10) and *SB* (46). Overall, *PS* displayed a transposition activity comparable with *SB* (Figure 4E), and a typical OPI phenomenon, which is also observed for the *SB*, *ZB* and *PB* transposons (10,26)

### Cargo capacity of *PS* transposons

**Figure 5.**
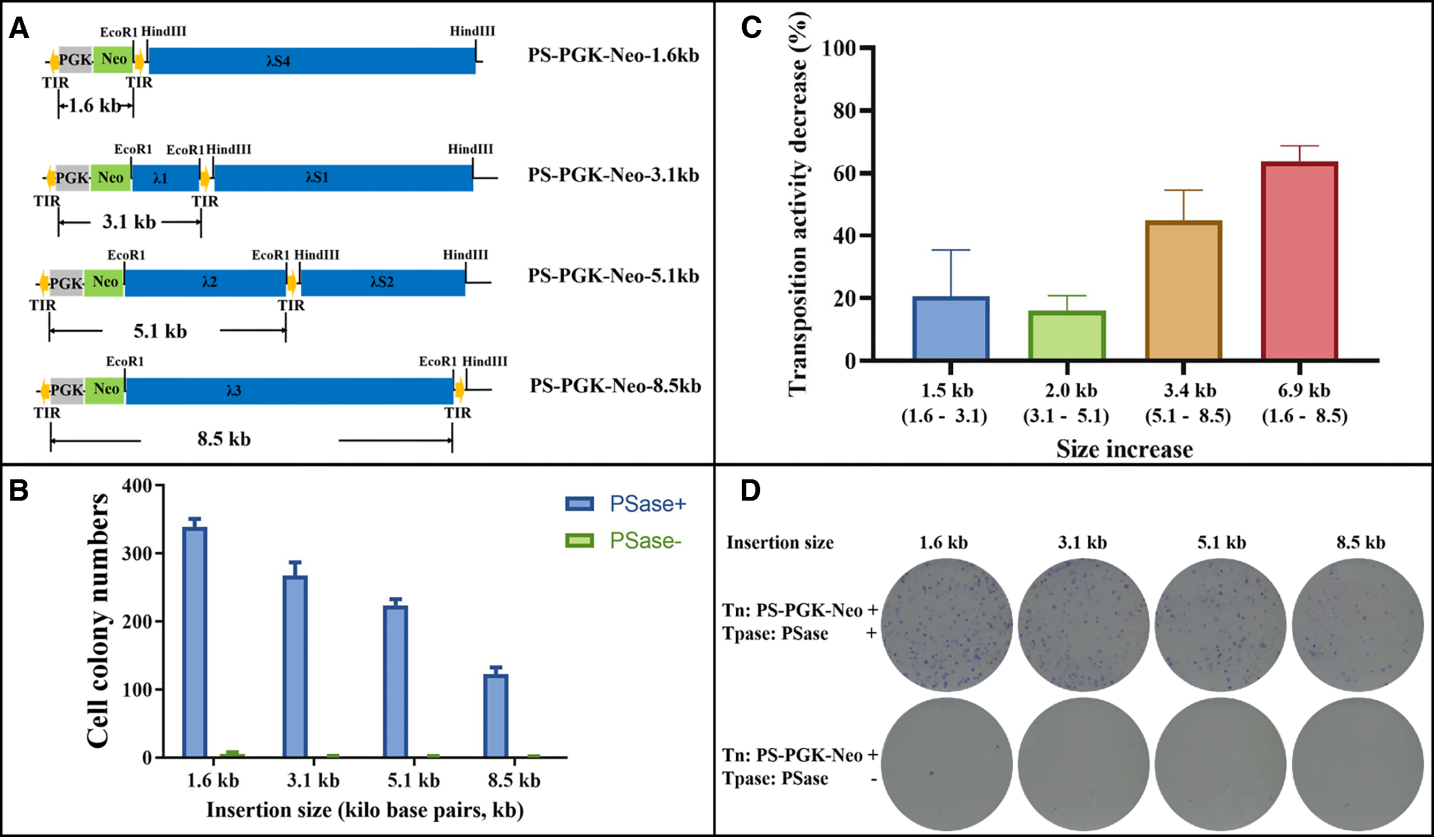
Cargo capacity of *PS* in human cells. (**A**) Donor plasmids used in human cells harbouring different sized fragments. Donor plasmids: the yellow arrows represent PSTIRs; *PGK*, *PGK* promoter; *Neo*, neomycin resistance gene. A DNA fragment with a size of λ kb (λ1, λ2, λ3 = 1.5, 3.5 and 6.9 kb) and another fragment of λS kb (λS1, λS2, λS4 = 5.5, 3.5 and 6.9 kb) were used as inner and outer stuffer fragments, respectively. (**B**) Comparative transposition activities of *PS* in HeLa cells co-transfected with different donor plasmids. (**C**) The transposition activity decreases with insertion size increases based on the data from (B). (**D**) G418-resistant cell colonies stained by Giemsa. The errors were calculated, and the bars (B and C) represent the results of three independent repeats.

Four donor plasmids harbouring insertions ranging in size from 1.6, 3.1, 5.1 to 8.5 kb between the TIRs (Figure 5A) were co-transfected with the helper plasmid (pcDNA3.9-PSase) into HeLa cells, to evaluate the cargo capacity of the *PS* system. The results showed that the transposition efficiency of the *PS* system decreased with the increase in the size of the inserted fragments (Figure 5B). The highest transposition activity was observed for the donor plasmid with the shortest fragment (1.6 kb), whereas the lowest colony numbers were observed for the donor plasmid containing the longest fragment (8.5 kb) (Figure 5B, C, D). The transposition activity decreased by ∼60% when the insertion size increased from 1.6 kb to 8.5 kb (Figure 5C); on average, the transposition activity of PS is decreased by ∼12% per kb increase of the insertion size, whereas in the *SB* system, the transposition efficiency was decreased by ∼30% per kb increase in the insertion size (45).

### Analysis of *PS* insertion sites in the human genome

**Figure 6.**
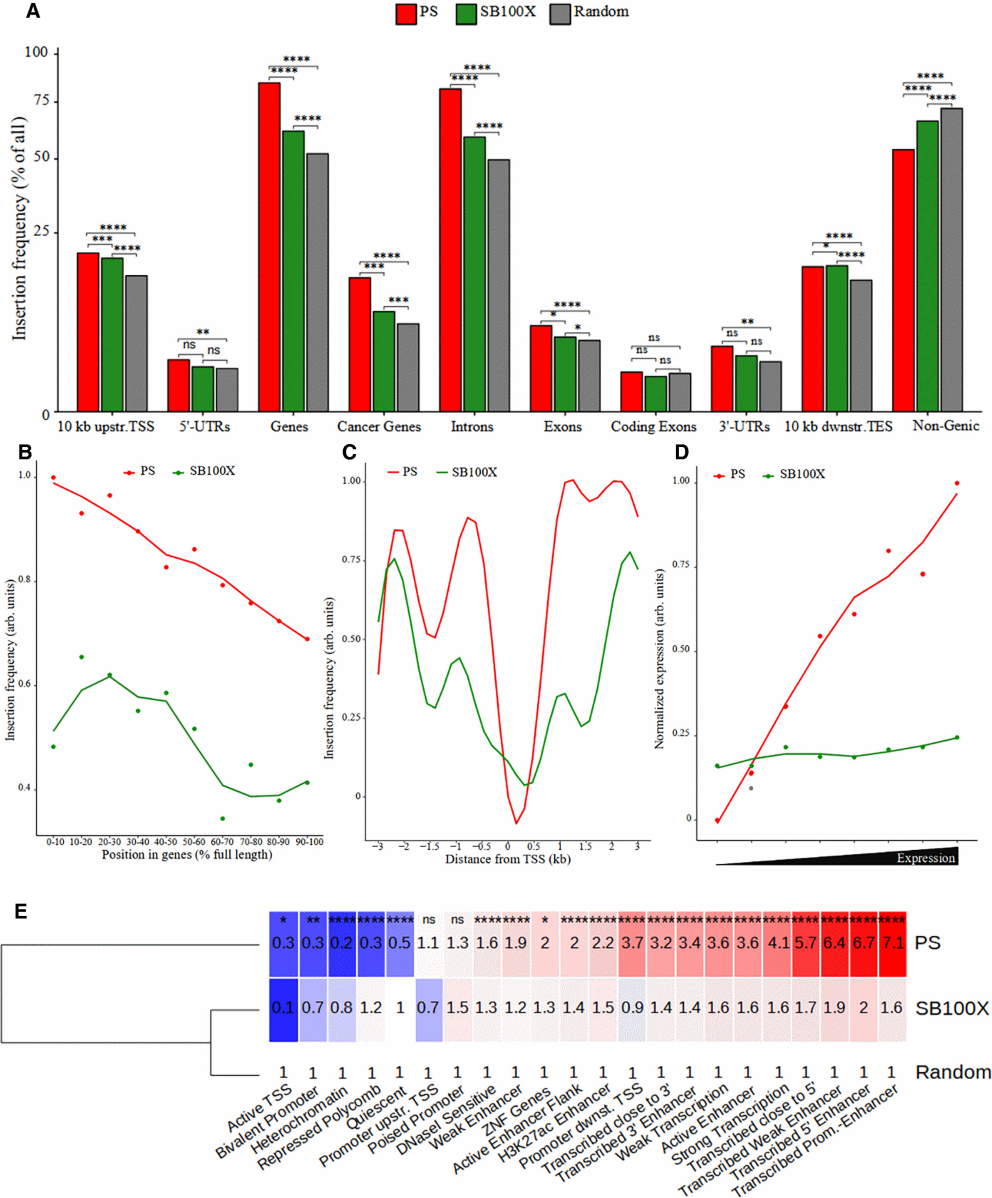
Analysis of the *PS* insertion sites in the human genome. (**A**) Distribution of *PS* and *SB* insertions in gene-related regions of the human genome. The *y*-axis is shown in a square root scale. The asterisks depict the significance ranges of Fisher's exact test. TSS, transcriptional start site; TES, transcriptional end site. (**B**) Distribution of insertion sites within gene bodies. For the analysis, all genes were divided into 10 segments of equal length; 100% stands for the last nucleotide of the genes. (**C**) Insertion site distributions in a 6 kb window centred on the TSSs of genes. (**D**) Integration frequencies in eight gene groups with increasing expression levels in HepG2 cells. (**E**) Insertion site frequencies in functional genomic categories. The categories on the *x*-axis were established using combined histone modification based on ChIP-Seq, RNA-Seq and DNase-Seq datasets for the HepG2 cell line. The numbers in the boxes are fold change values above the random expected frequencies (arbitrarily set to 1). The dendrogram on the left is based on row means. The red and blue colours stand for over- and under-representation, respectively. The asterisks indicate the significance ranges based on the *P*-values of Fisher's exact test between the values of *PS* and SB100X.

The *PS* transposons integrated into TA target dinucleotides situated at the centre of the AAGTACTT palindromic consensus sequence, surrounded by generally AT-rich DNA (Supplementary Figure S6). Next, we investigated the genomic distribution of the *PS* insertions and compared it with that of *SB* and a hypothetical random insertion distribution in human cells. We found that *PS* shows a preference for genic regions (Figure 6A). Analyses of the frequencies of insertions in gene bodies showed that *PS* integrations have a bias toward the 5′ end of the genes (Figure 6B) in human hepatocyte-derived HepG2 cells. In contrast, the less common genic insertions of *SB* tend to be located in gene bodies. In agreement with this finding, investigation of the frequencies of *PS* integrations around TSSs showed that *PS* was inserted upstream and downstream of TSSs more frequently than *SB* (Figure 6C). The study of the distribution of *PS* insertions within genes showed that higher expression was associated with a higher probability that a gene would be targeted by a *PS* transposition (Figure 6D). These results imply that *PS* exhibits a greater bias toward genes than *SB*, and that the chromatin structure associated with transcriptional activity may have an effect on the target site selection of the *PS* transposition machinery. Therefore, we used a 25 state HepG2 genome-specific chromatin state model, which was created based on ChIP-Seq, DNase-Seq and RNA-Seq data (47), and studied the insertion frequencies in these functional genomic segments. This analysis demonstrated elevated *PS* insertion frequencies in promoter and/or enhancer regions when these chromatin segments were associated with expressional activity and the activation of histone marks close to the 5′ end of transcripts (Figure 6E). Concomitantly, the representation of *PS* insertions in transcriptionally inactive chromosome segments and in heterochromatic regions was smaller than that of *SB*, or than that expected by random chance.

### *POGK* and *POGZ* derived from *PS* transposases do not display transposition activity

**Figure 7.**
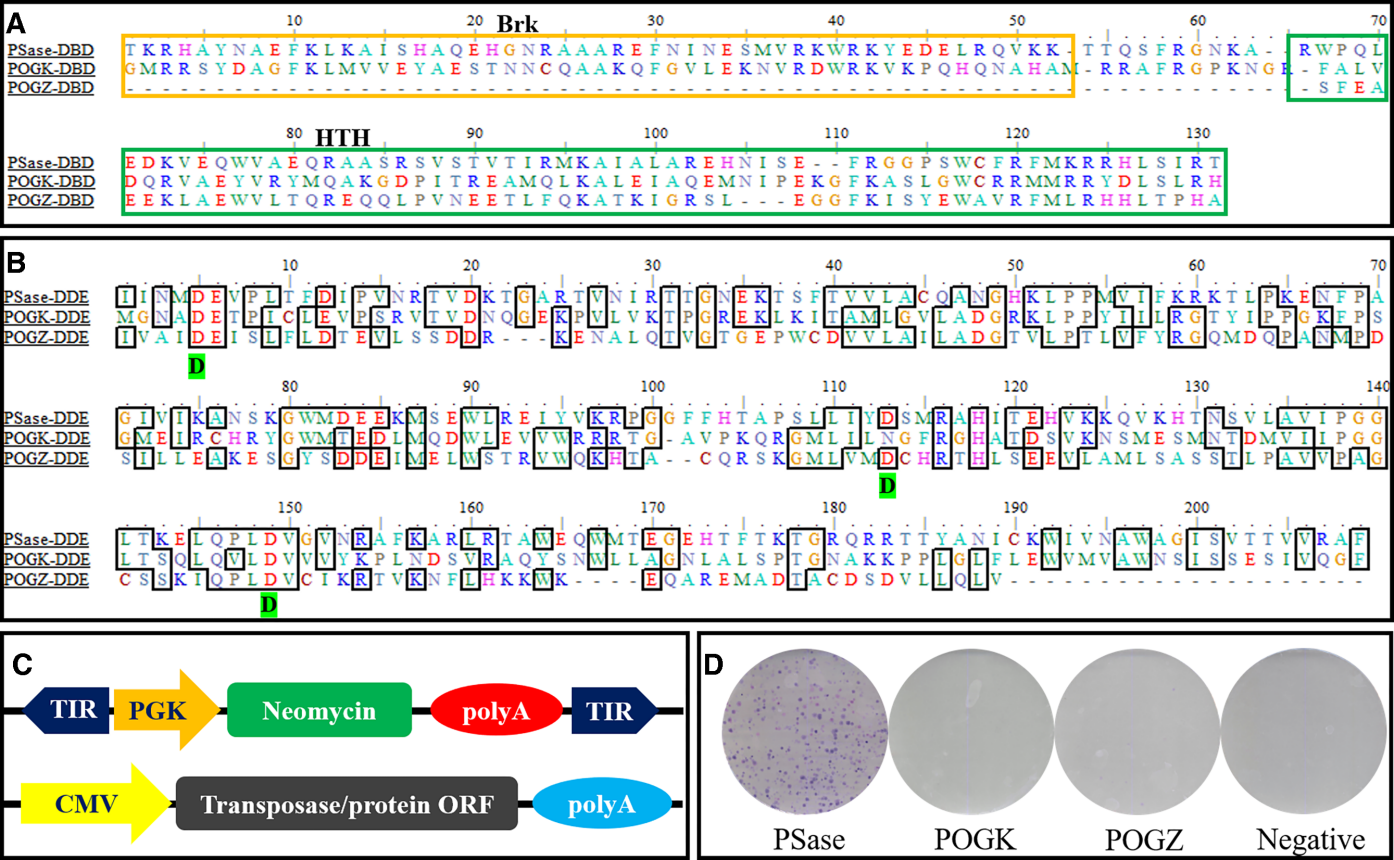
Transposition activities of *POGZ* and *POGK* in human cells. (**A**) DBD alignments of PSase, *POGK* (*pogo* transposable element derived with a KRAB domain, XP_005245427) and *POGZ* (pogo transposable element with a ZNF domain, NP_055915). (**B**) The alignments of the DDE domain from PSase, *POGK* and *POGZ*. (**C**) Donor plasmids: the blue arrows represent transposon TIRs; *PGK*, *PGK* promoter; *Neo*, neomycin resistance gene. Helper plasmids: *CMV*, *CMV* promoter; transposase, the ORFs of *PSase*, *POGK* and *POGZ* genes. (**D**) G418-resistant cell colonies stained by Giemsa.

The evolutionary histories of two domesticated genes of *pogo*-like transposons (*pogo* transposable element derived with a KRAB domain, *POGK*; and *pogo* transposable element with a ZNF domain, *POGZ*) have been well defined (4). They were domesticated from *PS* transposases and emerged in egg-laying mammals (Monotremata) and lobe-finned fish (Sarcopterygii) (4). Both *POGK* and *POGZ* have complete catalytic domains, similar to a canonical *PS* transposase (Supplementary Figure S7), and the triad signatures of the DD35D domain are well conserved in *POGZ*, while the second D of *POGK* is replaced by N. Furthermore, *POGK* contains two DBD motifs, while *POGZ* only carries one predicted helix–turn–helix (HTH) DBD motif (Figure 7A, B). Based on the homology and similarities among the three elements, we tested whether human *POGK* and *POGZ* could carry out transposition of the *PS* element from the donor plasmid (PS-PGK-Neo) into the human genome in HeLa cells. Upon transfection of the *POGK* and *POGZ* expression vectors into human HeLa cells together with PS-PGK-Neo (Figure 7C) and subsequent G418 selection, no colonies were obtained (Figure 7D), which was similar to what was observed for the negative control, suggesting that the *POGK* and *POGZ* genes have lost their capacity for transposition.

## DISCUSSION

Generally, two approaches have been applied to identify active transposons. One consists of mining of active elements in their natural forms by bioinformatics analysis, such as *PB*, *ZB*, *TcBuster*, *Tol2*, *Ac*/*Ds* and *Hobo* (10,22,48–51); and the other consists of the reconstruction of active elements from dead transposons found in nature, such as *SB*, which was molecularly rebuilt from a dead transposon family present in several fish genomes (52) and subsequently refined into a hyperactive version (SB100X) (53). In addition, the transposition activities of both *PB* and *TcBuster* were significantly improved by molecular optimization based on the original active transposase (54,55). *PS* transposons, which were defined as members of the *pogo* superfamily and display extensive distribution in animals and chromista, are characterized by a simple structural organization with short TIRs (<30 bp) and a single ORF encoding ∼400–500 amino acids, thus facilitating vector engineering for genetic manipulation. Here, evolution dynamics and sequence analyses suggested that *PS* elements in some species may be highly active. Furthermore, cell functional assays revealed that the native *PS* transposases from *G. aculeatus* and *D. rerio* displayed very high activity in human cells. In particular, *PS* transposons in *G. aculeatus* displayed a transposition activity comparable with that of *SB* (87%), which also agrees with the results of the bioinformatics analysis, where *PS* exhibited very high sequence identities and displayed very low K divergence in the genome of *G. aculeatus*, indicating that it is a very young invader in this species.

Here, we also characterized the three key biological features (OPI, cargo capacity and integration site preferences) of *PS* transposons, which will provide an important reference for the application of this transposon. OPI has been observed for a wide variety of transposons, including *SB* (56), *ZB* (10), *Tc1*/*mariner* (57), *PB* (58) and *Tol2* (26,59). It is thought that overoccupancy of the transposon ends with transposase dimers blocks assembly of the transpososome, which leads to the OPI phenomenon (60). Here, we demonstrated that the *PS* transposon showed the typical OPI phenomenon under conditions of high (500 ng) or low (10 ng) dosages of donor plasmids in HeLa cells, similar to the *SB* control (Figure 4B, C). However, *SB* seemed to be more sensitive to OPI than *PS*, as the activities of *PS* peaked at 250 and 500 ng of transfected transposase plasmid in the presence of a low (10 ng) and high (500 ng) amount of donor plasmid, respectively, whereas *SB* reached its peak activities at 50 ng of transfected transposase plasmid in the presence of a low (10 ng) and high (500 ng) amount of donor plasmid, respectively; this phenomenon has been described in the previous literature (26,27). These data indicate that the OPI may be common for *Tc1/mariner* and *hAT* DNA transposons, but the ratio (dosage) of donor and helper plasmids that is necessary to reach peak activity is substantially different across different transposon systems. In addition, we found that the plasmid vector sequences may have substantial impact on the OPI of *SB*; more obvious OPIs of *SB* were observed for the donor vector containing the SV40 promoter and the helper vector with the CAAGS promoter (Supplementary Figure S5), compared with the donor vector harbouring the PGK promoter and the helper plasmid carrying the CMV promoter (Figure 4C), which warrants attention for the OPI analysis of *SB* and the other transposons.

Cargo capacity plays an important role in transposition, which restricts the application of the transposon system for the generation of mutagenesis or for human gene therapy purposes (18,24). The tolerance for cargo size varies greatly between transposons, and the transpositional activity is generally limited by the increasing insertion size. The activity of *SB* has been shown to be inhibited by the increasing insertion size (45), although larger transposons can be more efficiently mobilized by hyperactive *SB* transposases and a ‘sandwich’ transposon vector (61). Both *SB* and *PB* transposons were shown to support transposition of inserts of >100 kb in size represented by bacterial artificial chromosomes (BACs) (62), whereas *Tol2* was used to deliver foreign genes of ∼70 kb to the zebrafish and mouse genomes successfully (63). A decrease in transposition efficiency with increasing cargo size was observed for *PS* transposons, with the transposition activity observed for an 8.5 kb insertion size being lower than that detected for 1.6 kb. Although *PB* (48) and *Tol2* (64) transposons appear to be more tolerant to increasing cargo size, it is still worth developing *PS* transposons as genetic tools with relatively high activity for different purposes. Furthermore, the cargo capacity of *PS* could be improved after optimizing the TIR structure based on experience with *SB* (56).

Integration site preference can greatly affect the utility of transposon systems for different applications (8). For instance, transposon systems for human gene therapy should satisfy the requirements of exhibiting the least preference for target genes. In contrast, transposons exhibiting a preference for a location in genes and gene-regulatory regions might work better for mutagenesis. On a genomic scale, *PB*, *Tol2*, *TcBuster*, *SPIN* and *ZB* transposons have a bias to insert into transcriptional regulatory regions of genes (10,22,54,65,66), while a close to random integration profile was observed for the *SB* transposon (65), indicating that *TcBuster*, *SPIN*, *PB*, *ZB* and *Tol2* are more suited than *SB* for application in functional genomic analysis (such as gene and enhancer trapping), whereas *SB* may be safer than other transposon systems for gene therapy. Here, *PS* exhibited the highest propensity for transcriptional regulatory regions and genes among others, indicating that *PS* may possess the potential for functional genomic analysis.

Recurrent domestications of DNA transposons in mammals, including humans, have been reported extensively (67,68). At least 12 protein-coding genes domesticated from the DD × D/*pogo* superfamily in vertebrates were identified, and they were derived from the different *pogo* transposase families. Phylogenetic analysis revealed that *POGK* and *POGZ* were derived from *PS* transposases (4). Transposition activity may remain for some transposase domesticated genes in the human genome, which may induce transposition or other genomic rearrangements. Indeed, transposition activity has been observed for the *P*-element-derived gene *THAP9* in the human genome (69). Enzymatic activity of the human *piggyBac* transposable element derived 5 (PGBD5) protein has been suggested as an oncogenic mutator which may be involved in site-specific DNA rearrangements in childhood and adult solid tumours (70,71), although contradictory results on PGBD5 transposition have been reported (72). In turn, the recombination-activating gene 1 (RAG1) and RAG2 proteins, which are derived from the *Transib*-like DNA transposase, belong to the lymphoid-specific factor family and catalyse the somatic recombination of the Ig and T-cell receptor genes in lymphocytes (73), also maintain transposition activity *in vitro* (74) and have been suggested as another possible source of oncogenic mutators (75,76). *POGZ* has been associated with intellectual disability and autism spectrum disorders (77,78), and plays a role in modulating chromatin structure (79); however, the biological role of *POGK* is unknown. Here, we provided evidence to support the contention that the two *PS*-derived genes (*POGK* and *POGZ*) have lost the transposition activity in human HeLa cells; however, it is unclear whether their lost jumping activity is caused by the DBDs, or that the catalytic domains (DDE) of *POGK* and *POGZ* are not functional, warranting further evaluation.

In summary, the current study demonstrated that *PS* is a noticeably young invader of the *G. aculeatus* genome that displayed high transposition activity in mammalian cells and a differential genome-wide integration preference compared with *SB*. Therefore, *PS* transposons represent alternative, powerful genetic tools for transgenesis and insertional mutagenesis in vertebrates.

## DATA AVAILABILITY

All data needed to evaluate the conclusions in this paper are present either in the main text or in the supplementary data.

## Supplementary Material

gkad005_Supplemental_Files
