# Supplementary material for: Passer, a highly active transposon from a fish genome, as a potential new robust genetic manipulation tool
Source: Nucleic Acids Res. 2023 Jan 23;51(4):1843–58. doi: 10.1093/nar/gkad005 (PMC9976928; doi:10.1093/nar/gkad005)
Supplement: gkad005_Supplemental_Files [file gkad005_supplemental_files.zip › Supplementary Figure 22-12-26.pdf]

A

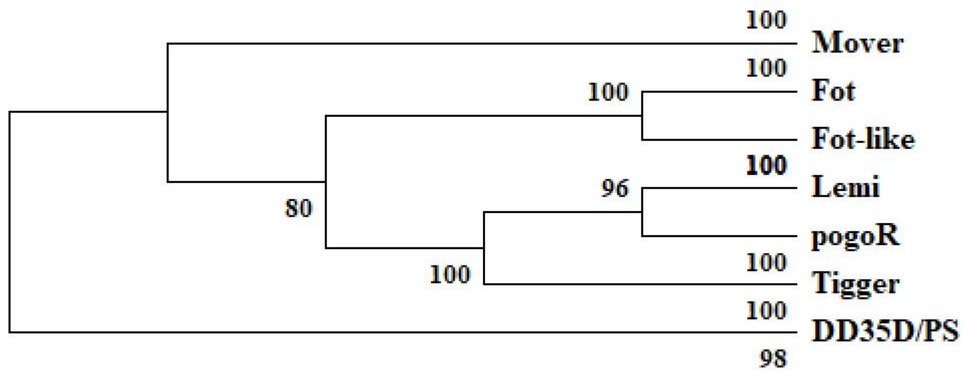

B

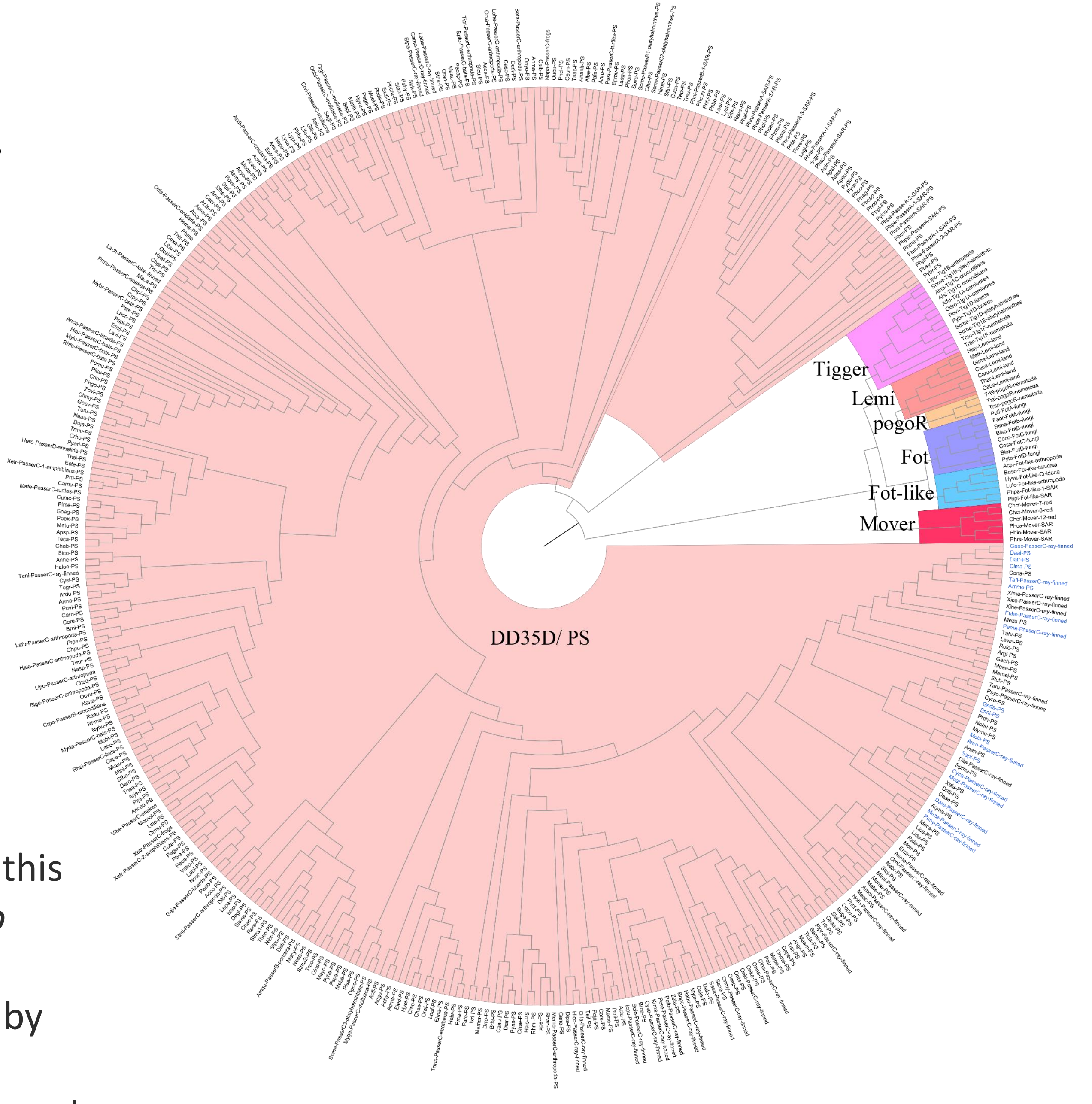

Supplementary Fig. S1 (**A**) Phylogenetic tree of *PS*s identified in this study and previous reported with six other members of the *pogo* superfamily based on their DDD/E domain of transposases. Bootstrapped (1000 replicates) phylogenetic trees were inferred by using the maximum likelihood method in IQ-TREE. (**B**) the unfold form of phylogenetic tree. TIRs from 18 species (blue font) were used to construct the consensus TIR sequence.

A

Taxa distribution	Motif	Number of species	Species containing intact copy	Length of full TN (bp)	Length of TPase (aa)	Length of TIR (bp)	TSD
Stramenopiles	DD35D	40	24	711-2334	203-568	18-29	TA
Rhizaria	DD35D	1	0	957	319	-	
Porifera	DD35D	2	1	2207-2557	449-459	26-149	TA
Cnidaria	DD35D	31	20	510-3396	124-473	18-28	TA
Platyhelminthes	DD35D	3	3	1697-2163	330-446	21-28	TA
Mollusca	DD35D	26	10	720-3200	124-488	21-28	TA
Annelida	DD35D	4	1	762-1773	254-437	24	TA
Nematoda	DD35D	9	3	693-2435	111-502	21-54	TA
Echinodermata	DD35D	5	1	678-4073	226-431	25	TA
Urochordata	DD35D	3	1	1522-2903	196-430	23-26	TA
Cephalochordata	DD35D	1	0	1293	431	-	
Chondrichthyes	DD35D	6	1	834-3015	121-497	24-28	TA
Testudines	DD35D	16	8	900-2704	102-462	19-36	TA
Squamata	DD35D	24	16	468-4078	156-450	18-29	TA
Crocodilia	DD35D	14	4	579-2728	193-516	19-28	TA
Monotremata	DD35D	2	1	1556-1704	475-522	20	TA
Rodentia	DD35D	3	1	1257-2348	419-519	26	TA
Insectivora	DD35D	1	0	1275	425	-	
Chiroptera	DD35D	25	13	1314-3621	106-541	12-27	TA
Cetartiodactyla	DD35D	3	0	1014	338	-	
Anura	DD35D	12	10	1677-2781	178-446	15-28	TA
Arthropoda	DD35D	41	20	789-3379	186-506	19-38	TA
Afrotheria	DD35D	9	1	1296-2744	394-466	18-27	TA
Sarcopterygii	DD35D	1	1	1999	425	28	TA
Actinopterygii	DD35D	111	55	489-3453	142-503	10-31	TA

B

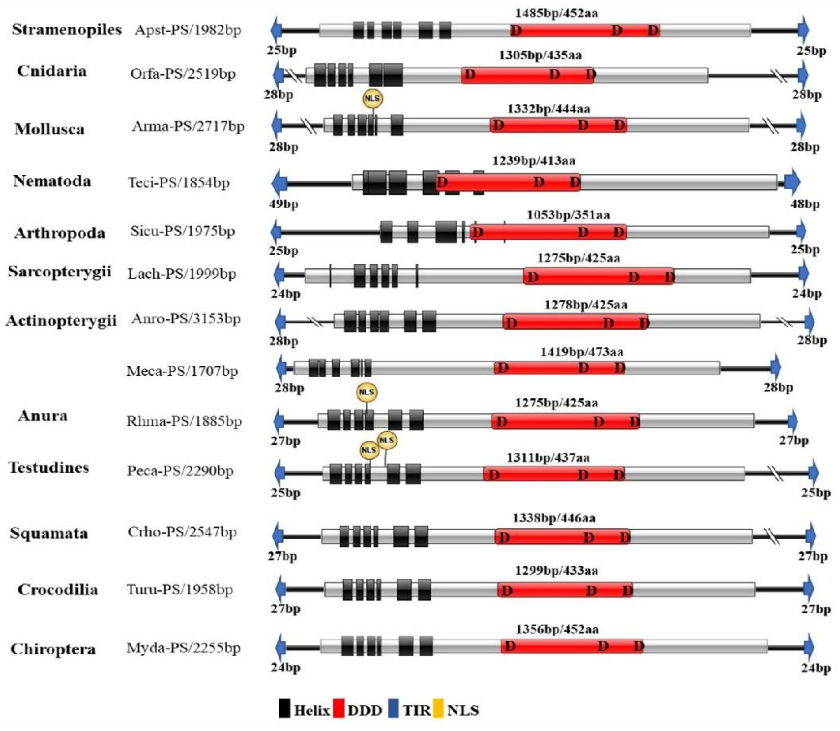

Supplementary Fig. S2 (**A**) Summary of total information of *PS* family in the kingdom. (**B**) The structural and functional component of *PS*s in representative species. Blue arrow, TIR; Black rectangle, helix; Red rectangle, DDE; Yellow bubbles, NLS.

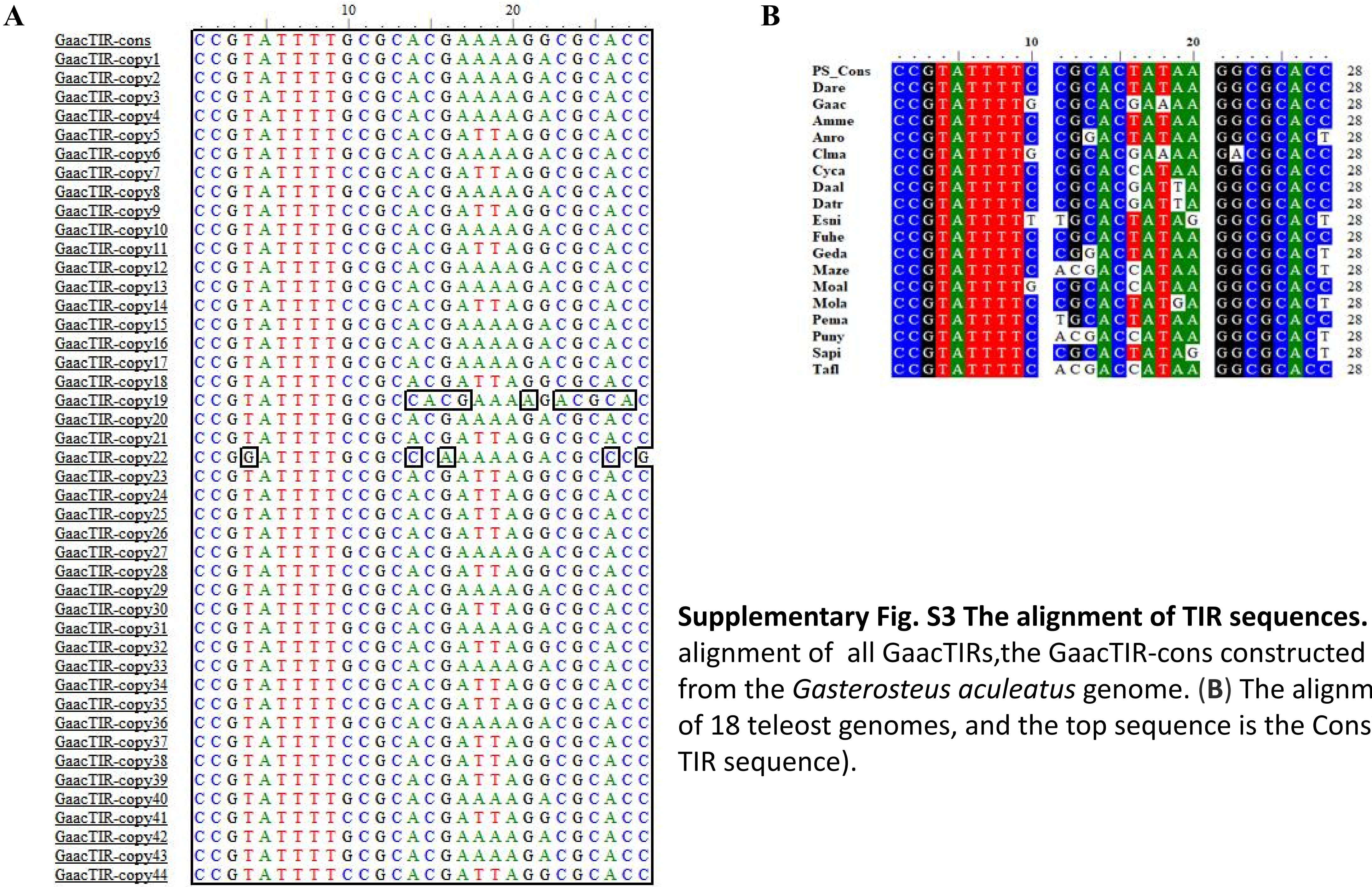

Supplementary Fig. S3 The alignment of TIR sequences. (**A**) The alignment of all GaacTIRs,the GaacTIR-cons constructed using all PS TIRs from the *Gasterosteus aculeatus* genome. (**B**) The alignment of the PSTIRs of 18 teleost genomes, and the top sequence is the ConsTIR (consensus TIR sequence).

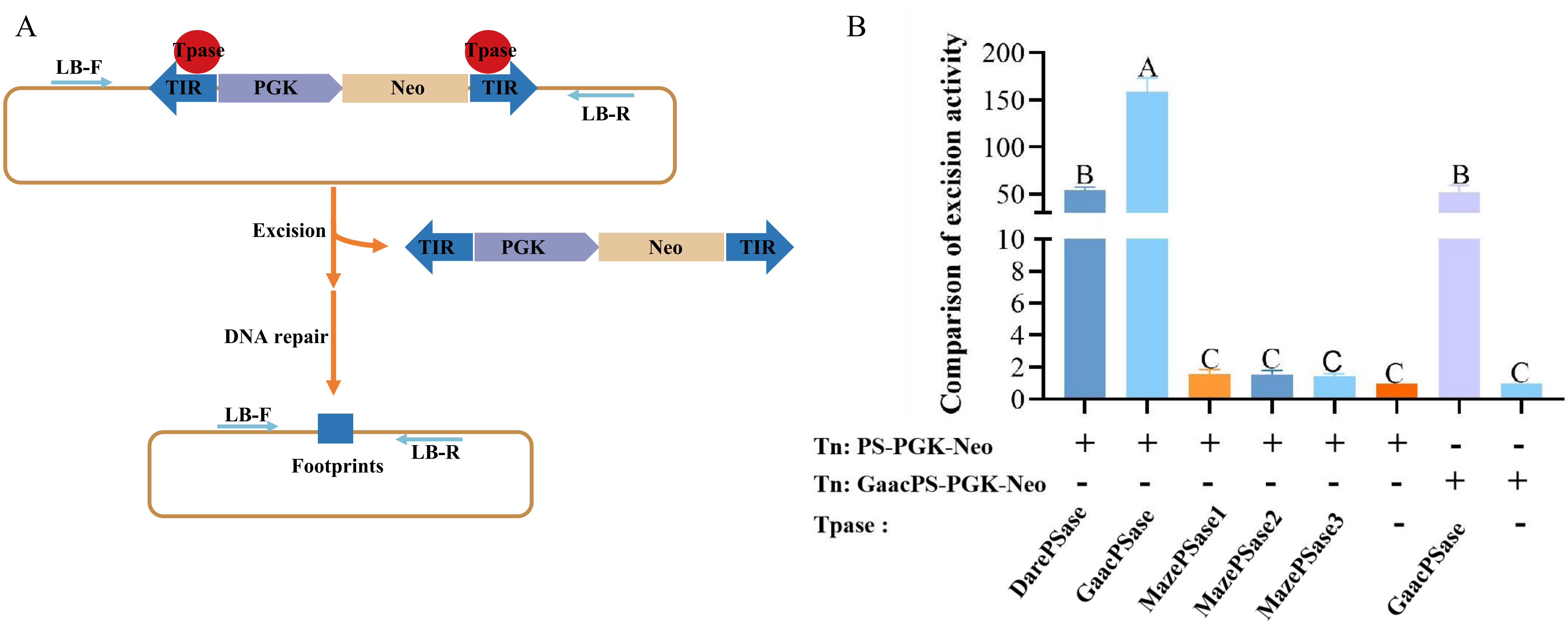

Supplementary Fig. S4 (**A**) Excision process of *PS.* A schematic representation of the donor is shown on the top. The footprints would be obtained by sequencing. (**B**) Comparative of excision efficiency of different *PS* groups via qPCR. Different capital letters showed significant difference ($p<0.01$) between bars, while the same capital letters showed no difference ($p>0.05$) between bars.

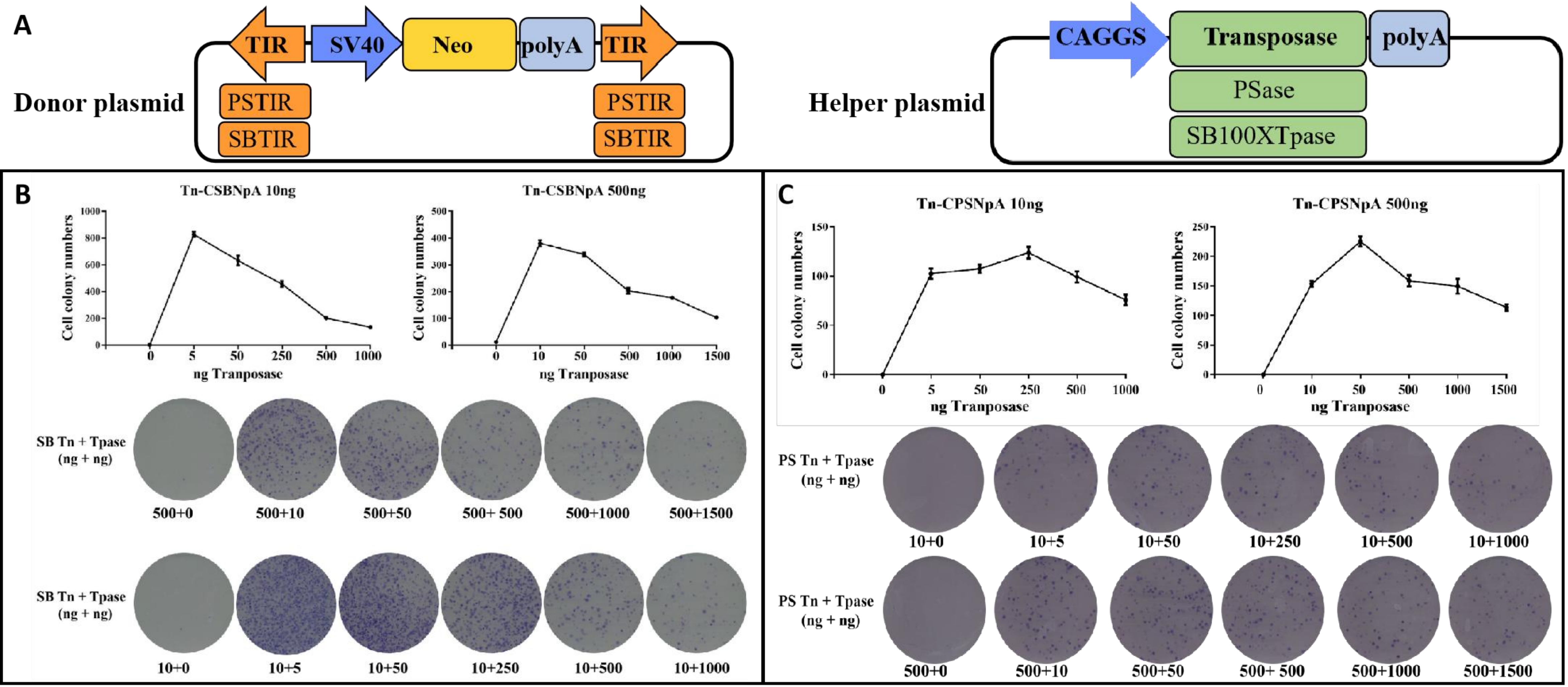

Supplementary Fig. S5 Transposition activities of PS and SB by using reported donor vector backbone (27) in human cells. After tansfection 48h, 1×10^5 transfected cells were transferred into 10cm plates for 10 ng of donor plasmid group, and 1×10^4 transfected cells were seeded into 10cm plates for 500 ng of donor plasmid group. (**A**) Donor and helper plasmids used in human cells. Donor plasmids: the arrows represent transposon terminal inverted repeats (TIRs); *SV40*, SV40 promoter; *Neo*, neomycin resistance gene. Helper plasmids: *CAGGS*, CAGGS promoter; transposase, the transposase (PSase and SB100X)-encoding gene. (**B**)Transposition activities of SB in HeLa cells co-transfected with different transposon DNA conditions (10 and 500 ng). (**C**) Transposition activities of PS in HeLa cells co-transfected with different transposon DNA conditions (10 and 500 ng).

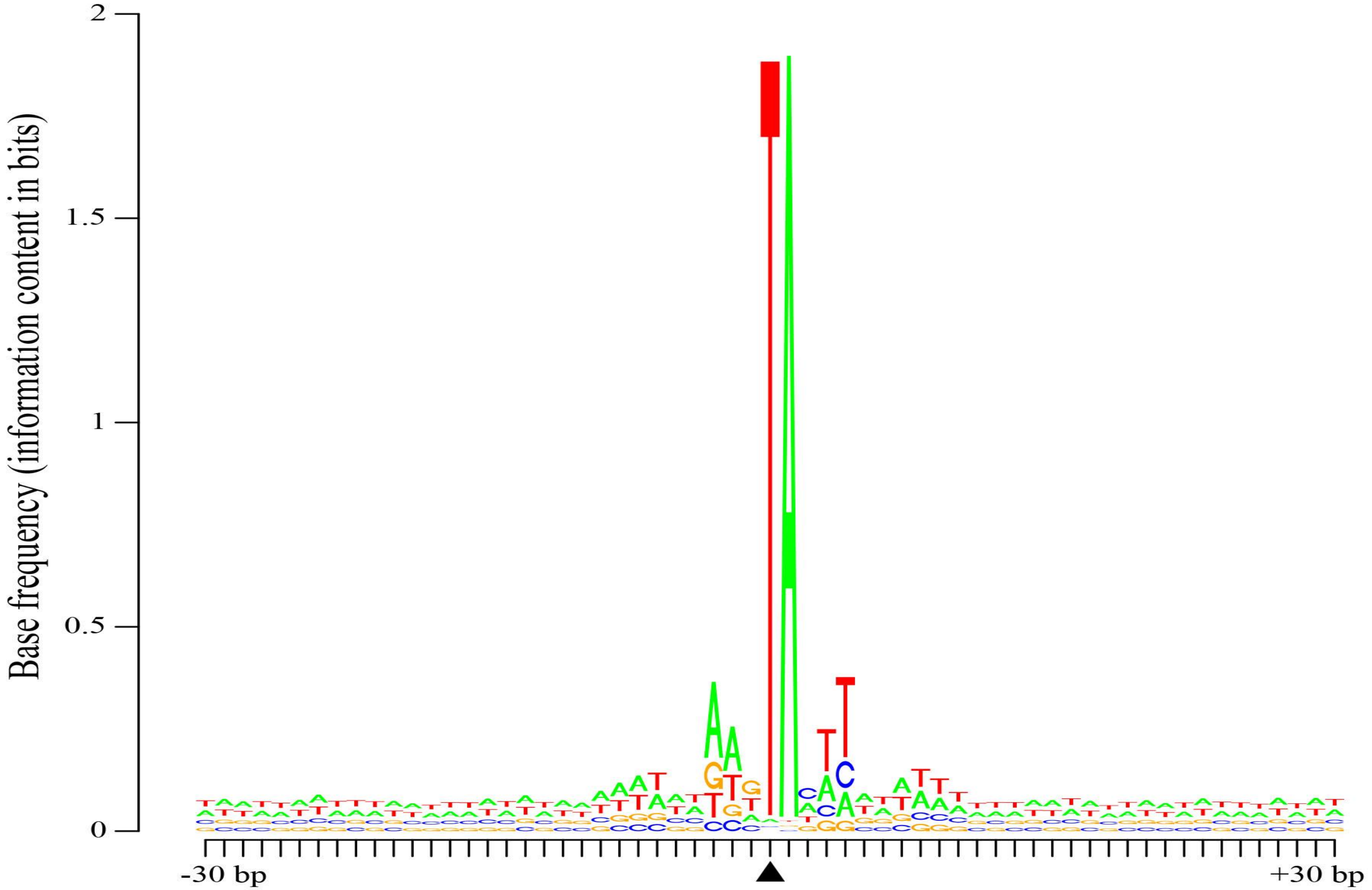

Supplementary Fig. S6 Base representation of nucleotides at the genomic insertion sites of *PS*. The sequence logo shows the frequency of bases at the insertion sites in a 60-nucleotide window, centered on the integration locus (black arrow). The relative sizes of the letters indicate their frequency in the sequences on the y-axis (2 stands for 100% conservation).

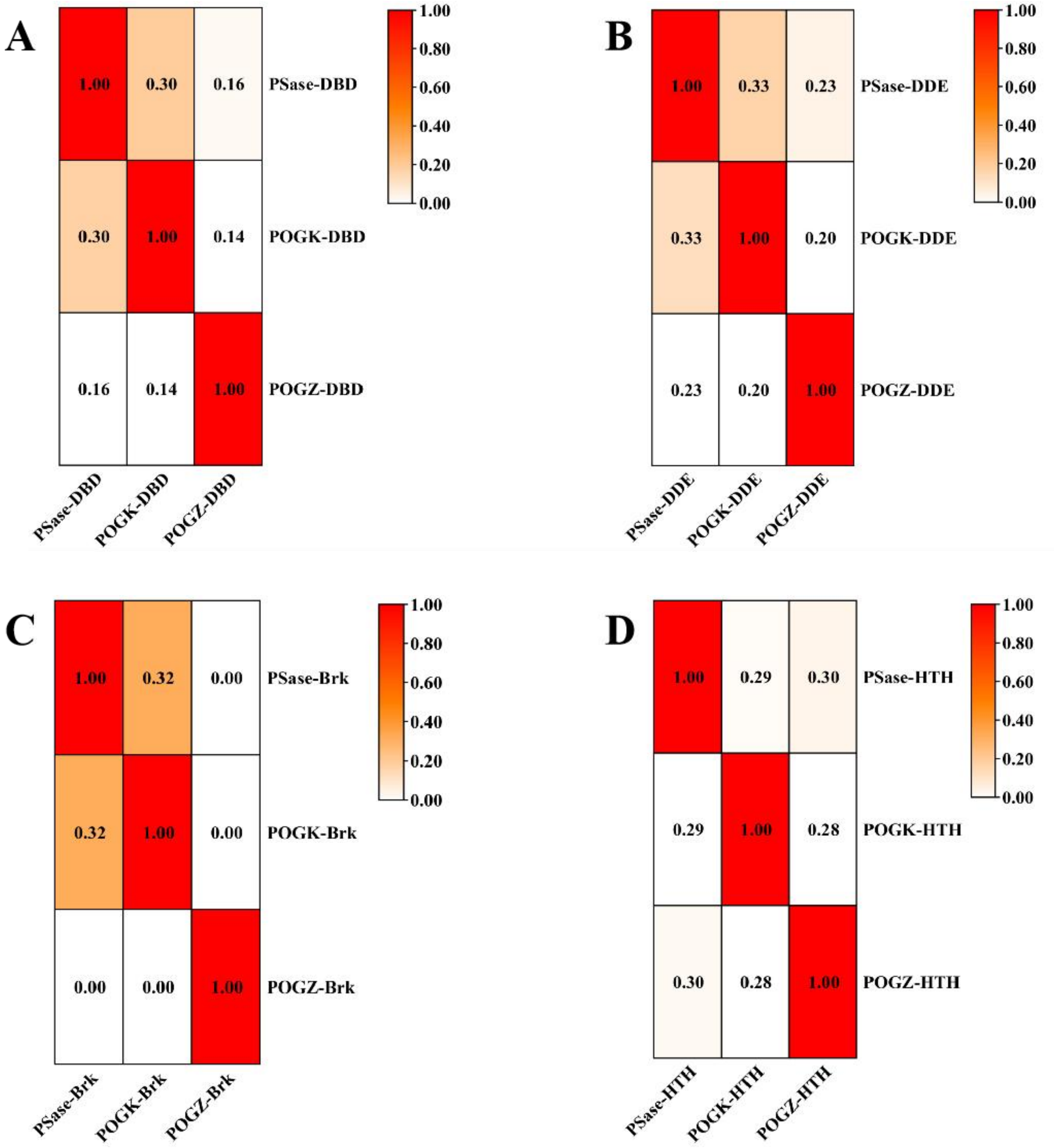

Supplementary Fig. S7 Sequence identities of DBD and DDE domains among three proteins (*PSase*, *POGK*, *POGZ*), which were measured by pairwise comparison. The numbers in the heatmap are the sequence's identities of the two protein sequences in the corresponding row and column (**A-D**). The values of sequence identities were measured by pairwise comparison of the whole DBD (**A**), DDE (**B**), Brk (**C**) and HTH (**D**) DBD domains.
