# Supplementary material for: Passer, a highly active transposon from a fish genome, as a potential new robust genetic manipulation tool
Source: Nucleic Acids Res. 2023 Jan 23;51(4):1843–58. doi: 10.1093/nar/gkad005 (PMC9976928; doi:10.1093/nar/gkad005)
Supplement: gkad005_Supplemental_Files [file gkad005_supplemental_files.zip › Supplementary Table S2. Primers used in this assay.docx]

**Supplementary Table S2. Primers used in this assay.**

| Primer | Sequence(5’-3’) |
| --- | --- |
| PS-TIR-F | GGATCCTACCGTATTTTCCGCACTATAAGGCGCACCTCGCGAGCGGCCGCGAATTC |
| PS-TIR-R | GGATCCTACCGTATTTTCCGCACTATAAGGCGCACCGAATTCGCGGCCGCTCGCGA |
| GAAC-TIR-F | GGATCCTACCGTATTTTGCGCACGAAAAGGCGCACC TCGCGAGAATTC |
| GAAC-TIR-R | GGATCCTACCGTATTTTGCGCACGAAAAGGCGCACCGAATTCTCGCGA |
| Gaac-flank-F | GGTCTCTGTTGCTCTCTGGT |
| Gaac-flank-R | CGCTGTATGAAACGATGCCA |
| Gaac-CDS-F | GGATCCGCCACCATGGCTCCTACCAAAAGACA |
| Gaac-CDS-R | CTCGAGCTAAAATCCCTCGAAAACTT |
| Dare -CDS-F | aaACTAGTGCCACCATGGCACCAATGAAGAGACA |
| Dare -CDS-R | aaACGCGTTCAGTCTTCTCCCACAAATC |
| Maze -22-flank-F | AGCGATCAGGTAGCACATCA |
| Maze -22-flank-R | ACAGCACAAATCCGATACGC |
| Maze -22-CDS-F | GGATCCGCCACC ATGTGCGTTATGCAGGACAT |
| Maze -22-CDS-R | GAATTCTCAATCAAACCCATCGAAGTCC |
| Maze -24- flank-F | ATACATGCAGGTAGGGCTGG |
| Maze-24- flank-R | CTCTCTGTCCTGGTCGTTGT |
| Maze -22-CDS-F | GGATCCGCCACC ATGTGCGTTATGCAGGACAT |
| Maze -22-CDS-R | GAATTCTCAATCAAACCCATCGAAGTCC |
| Maze -17- flank-F | AGCAGCAACTACCACAAAGC |
| Maze-17- flank-R | GCCTACCCTACTCCTGCTTT |
| Maze -17-CDS-F | GGATCCGCCACC ATGTGCATTATGCAGGACATCG |
| Maze -17-CDS-R | GAATTCTCAATCAAACCCATCGAAGTCC |
| SB100X-F | CGGGATCCCCATGGGAAAATCAAAAGAA |
| SB100X-R | CCCTCGAGCTAGTATTTGGTAGCATTGC |
| UC19PSneo-F | GGGGTACCtaccgtattttccgcactataaggcgcaccctggaattctgtggaatgtgtgtcagttagggtg |
| UC19PSneo-R | GCTCTAGAtaccgtattttccgcactataaggcgcaccggtacagacatgataagatacattgatgagtttg |
| PSase-F | CCCTCGAGGCCACCATGGCTCCTACCAAAAGA |
| PSase-R | TTgcggccgcCTAAAATCCCTCGAAAACTTCTTCT |
| λ1-F | CGGAATTCGGATGGTGATGCCGAGAACTTTATG |
| λ1-R | CGGAATTCCATTGCGTCGCTTTTTGCTGTC |
| λ2-F | CGGAATTCGGATGGTGATGCCGAGAACTTTATG |
| λ2-R | CGGAATTCGCTGGCAATATGCGGGAGATTAC |
| λ3-F | CGGAATTCGGATGGTGATGCCGAGAACTTTATG |
| λ3-R | CGGAATTCCCCGTCCAAGCCAGAGATGAC |
| λS1-F | CCAAGCTTAGGCACTCGACTGCTTCGTT |
| λS1-R | CCAAGCTTCCCGTCCAAGCCAGAGATGAC |
| λS2-F | CCAAGCTT ATGGCCTTTAATGAGCCGCT |
| λS2-R | CCAAGCTTCCCGTCCAAGCCAGAGATGAC |
| λ4-F | CCAAGCTT GGATGGTGATGCCGAGAACTTTATG |
| λ4-R  LB-F  LB-R | CCAAGCTT CCCGTCCAAGCCAGAGATGAC  CGACTCACTATAGGGAGAGCGGC  AAGAACATCGATTTTCCATGGCAG |
| Footprint-seq | GCAGGTTCCATTCATTGTTTTT |
| Cut129-F | CCGGCGTAATACGACTCACT |
| Cut129-R | TTCCATGGCAGCTGAGAATA |
| Amp104-F | TTGCCGGGAAGCTAGAGTAA |
| Amp104-R | AAGCCATACCAAACGACGAG |
| GFP cassette F | ccgtattttccgcactataaggcgcaccactagtgcttttagaccttcttacttttgg |
| GFP cassette R | ccgtattttccgcactataaggcgcaccTTAATTAAagcttgggctgcaggtcgag |
| LinkerNest1 | gtaatacgactcactatagggc |
| LinkerNest2 | caagcagaagacggcatacgagatNNNNNNgtgactggagttcagacgtgtgctct tccgatct (NNNNNN stands for the barcode sequence) |
| PSnest1 | ggtgcgccttatagtgcggaaa |
| PSnest2 | aatgatacggcgaccaccgagatctacactctttccctacacgacgctcttccgatcttgcgccttatagtgcggaaaat |
| POGZ-CDS-F | aaCCGCGGGCGTAATACGACTCACTATAGGGGCCACCatggcggacaccgac |
| POGZ-CDS-R | aaCCGCGGtcaaatctccatcagatcta |
| POGK-CDS-F | aaCCGCGGGCGTAATACGACTCACTATAGGGACTAGTGCCACCatggagtccacagcc |
| POGK-CDS-R | aaCCGCGGACGCGTtcagttgctctcagccatgc |
